# Extracellular vesicles carry SARS‐CoV‐2 spike protein and serve as decoys for neutralizing antibodies

**DOI:** 10.1002/jev2.12112

**Published:** 2021-06-18

**Authors:** Zach Troyer, Najwa Alhusaini, Caroline O. Tabler, Thomas Sweet, Karina Inacio Ladislau de Carvalho, Daniela M. Schlatzer, Lenore Carias, Christopher L. King, Kenneth Matreyek, John C. Tilton

**Affiliations:** ^1^ Center for Proteomics and Bioinformatics Department of Nutrition School of Medicine Case Western Reserve University Cleveland Ohio USA; ^2^ Division of General Medical Sciences School of Medicine Case Western Reserve University Cleveland Ohio USA; ^3^ Department of Pathology School of Medicine Case Western Reserve University Cleveland Ohio USA

**Keywords:** coronavirus, decoy, extracellular vesicle, neutralizing antibody, SARS‐CoV‐2, spike

## Abstract

In late 2019, a novel coronavirus named severe acute respiratory syndrome coronavirus 2 (SARS‐CoV‐2) emerged in Wuhan, China. SARS‐CoV‐2 and the disease it causes, coronavirus disease 2019 (COVID‐19), spread rapidly and became a global pandemic in early 2020. SARS‐CoV‐2 spike protein is responsible for viral entry and binds to angiotensin converting enzyme 2 (ACE2) on host cells, making it a major target of the immune system – particularly neutralizing antibodies (nAbs) that are induced by infection or vaccines. Extracellular vesicles (EVs) are small membraned particles constitutively released by cells, including virally‐infected cells. EVs and viruses enclosed within lipid membranes share some characteristics: they are small, sub‐micron particles and they overlap in cellular biogenesis and egress routes. Given their shared characteristics, we hypothesized that EVs released from spike‐expressing cells could carry spike and serve as decoys for anti‐spike nAbs, promoting viral infection. Here, using mass spectrometry and nanoscale flow cytometry (NFC) approaches, we demonstrate that SARS‐CoV‐2 spike protein can be incorporated into EVs. Furthermore, we show that spike‐carrying EVs act as decoy targets for convalescent patient serum‐derived nAbs, reducing their effectiveness in blocking viral entry. These findings have important implications for the pathogenesis of SARS‐CoV‐2 infection in vivo and highlight the complex interplay between viruses, extracellular vesicles, and the immune system that occurs during viral infections.

## 1 INTRODUCTION

In early January 2020, concerning reports emerged from Wuhan, Hubei Province, China of a respiratory illness causing pneumonia (Chan et al., 2020; Huang et al., 2020; Wang et al., 2020). The causative etiological agent was soon identified as a novel coronavirus, eventually named SARS‐CoV‐2 (Severe Acute Respiratory Syndrome Coronavirus 2), and the disease it causes was termed coronavirus disease 19 (COVID‐19) (4). SARS‐CoV‐2 is now known to be spread between people via the air, primarily in respiratory droplets and aerosols formed by actions such as speaking, coughing and sneezing (Kumar et al., 2020; Prather et al., 2020; van Doremalen et al., 2020; Zou et al., 2020). While many patients can have mild or asymptomatic infection, the mortality rate for COVID‐19 increases drastically with age and patients over 70 have a mortality rate nearing 20% (Li et al., 2020; Niu et al., 2020; TieLong et al., 2020; Wu & Mcgoogan, 2020). Common comorbidities such as obesity, diabetes, and hypertension also increase mortality rate, especially when present in combination (Richardson et al., 2020; Jain & Yuan, 2020). Due to a relatively high level of contagiousness, SARS‐CoV‐2 quickly spread throughout China and subsequently other countries across the world despite efforts to contain it (Li et al., 2020). On March 11th, the WHO declared COVID‐19 a global pandemic. As of December 7th, 2020, SARS‐CoV‐2 had infected over 67 million people across the globe, resulting in over 1.5 million deaths. In response to the health and economic consequences of the pandemic, an unprecedented scientific effort has been mobilized to develop vaccines and antiviral drugs, and several vaccine candidates have released encouraging preliminary data.

SARS‐CoV‐2 is not the first coronavirus to cause a concerning viral outbreak. In 2002, a coronavirus now called SARS‐CoV‐1 infected around 8000 people with a death rate approaching 10%, before disappearing from the human population in 2004 (Dowell & Ho, 2004; Rm et al., 2004). SARS‐CoV‐2, like its predecessor, is an enveloped positive‐sense single‐stranded RNA virus in the genus *Betacoronavirus* (Cui et al., 2019; Wu et al., 2020). It contains four structural proteins: nucleocapsid, envelope, membrane, and spike; the latter three are incorporated into the viral membrane (Cui et al., 2019; Naqvi et al., 2020). Spike is the protein responsible for receptor binding, uptake and membrane fusion allowing viral entry into infected cells, often epithelial cells in the respiratory tract (Hou et al., 2020; Letko et al., 2020; Walls et al., 2020). Spike assembles as a non‐covalent trimer on the surface of viruses and binds to angiotensin converting enzyme 2 (ACE2), a receptor it shares with SARS‐CoV‐1, leading to fusion between the viral and host cell membranes (Hoffmann et al., 2020; Walls et al., 2020). Spike, consisting of subunits S1 and S2, is incorporated on the outside of SARS‐CoV‐2 particles and is the primary target for antibodies elicited by infection or vaccination (Rogers et al., 2020; Walls et al., 2020). In particular, neutralizing antibodies (nAbs) that target the spike protein can prevent binding to ACE2 and thereby block fusion and infection. Viruses have evolved countermeasures to disrupt or evade nAbs, including interfering with antigen presentation and interferon signalling, ‘escape mutations’ in viral envelope or spike proteins that reduce neutralization efficiency, and even the production of defective or sub‐viral particles that act as ‘decoys’ by binding to nAbs and reducing the effective concentration of ‘free’ nAb available to neutralize infectious virions (Bailey et al., 2004; Joyner et al., 2011; Lazarevic et al., 2019; Rydell et al., 2017; Weber & Haller, 2007; Yewdell & Hill, 2002). These evasion strategies allow viruses to circumvent the host response and infect new cells even in the presence of a nAb response.

Both healthy and virally‐infected cells release extracellular vesicles (EVs): membrane‐bound particles ranging in diameter from 40 nm to 5 μM (Akers et al., 2013; Nolte‐‘T Hoen et al., 2016; Simons & Raposo, 2009). EVs have historically been classified by their sizes and path of release from the cell. Exosomes are small, 40–100 nm EVs that bud into multivesicular bodies (MVBs) within cells, and are released when the MVB fuses with the plasma membrane of the cell (Johnstone et al., 1987). Microvesicles are 100–1000 nm EVs that are believed to bud directly from the plasma membrane (Heijnen et al., 1999). Apoptotic bodies, fragments of cells undergoing programmed cell death, are the largest EVs and have a diameter between 1–5 μm (Kerr et al., 1972). Due to size overlap between these classes of EVs and a lack of consensus on markers to specifically identify EVs originating from distinct subcellular locations, it remains difficult to unambiguously classify EVs into exosomes, microvesicles, or apoptotic bodies. Due to this issue, and in accordance with the International Society for Extracellular Vesicles (ISEV) 2018 position statement (MISEV 2018), the particles in this study are simply termed ‘EVs’. (Théry et al., 2018)

EVs contain proteins—both transmembrane and free—as well as small nucleic acids like miRNAs that are reflective of their cells of origin (Abels & Breakefield, 2016). Through the shuttling of these protein and RNA cargos between cells, EVs have been shown to impact target cell cellular behaviour and are now appreciated to play major roles in intercellular communication networks (Becker et al., 2016; Cabral et al., 2018; Frühbeis et al., 2012). Interestingly, EVs share many characteristics with enveloped viruses: they are sub‐micron particles, enclosed in lipid membranes, comparable in size, and originate from similar subcellular compartments and utilize overlapping egress machinery from the cell (Homman‐Loudiyi et al., 2003; Mori et al., 2008; Nolte‐‘T Hoen et al., 2016). In particular, the endosomal sorting complex required for transport (ESCRT) machinery plays an important role in both EV and viral release from cells (Nolte‐‘T Hoen et al., 2016). Considering this substantial overlap, it is perhaps unsurprising that EVs released from virally‐infected cells have been shown to carry viral proteins and RNAs. For instance, EVs from HIV infected cells carry HIV‐1 transactivation response element (TAR) RNA, which stimulates the growth of cancer cells (Chen et al., 2018; Narayanan et al., 2013). Other studies have highlighted the ability of EVs to carry HIV‐1 Nef protein, an HIV virulence factor that facilitates viral infection by manipulating host cell machinery (Arenaccio et al., 2014; Lenassi et al., 2010; Shelton et al., 2012). In addition to soluble factors, EVs have been shown to incorporate viral spike or Envelope glycoproteins (often called fusion proteins) from a diverse range of viruses on their surface, including VSV‐G, Influenza HA, HIV‐1 gp120, and HCMV gB and gH (Arakelyan et al., 2017; Meyer et al., 2017; Testa et al., 2010; Zicari et al., 2018). The extent to which EV‐incorporated viral fusion proteins interact with the immune system and affect viral pathogenesis remains largely unexplored.

In this study, we used nanoscale flow cytometry, mass spectrometry, and immunoblot to demonstrate that SARS‐CoV‐2 spike protein is incorporated into purified EV populations. Importantly, we found that these spike containing EVs act as immune decoys, reducing the effectiveness of both commercial and convalescent patient nAb in blocking entry and infection by viruses bearing SARS‐CoV‐2 spike. Our findings suggest that EVs may play an important role in modulating antibody responses, and potentially patient outcomes, during SARS‐CoV‐2 infection and COVID‐19 disease. These findings highlight the complex biology of extracellular vesicles, viral proteins, and immune responses that occurs during viral infections.

## 2 MATERIALS AND METHODS

### 2.1 Cell lines and maintenance

HEK293T cells were obtained from the ATCC repository and grown in 225 cm^2^ tissue culture flasks in DMEM (Gibco) supplemented with 10% heat‐inactivated foetal bovine serum (HI‐FBS) and 1% penicillin/streptomycin. Stable hACE2‐HEK293T cells, which express hACE2 and miRFP670 under control of a doxycycline‐inducible promoter, were a kind gift from Dr. Kenneth Matreyek, generated using the landing pad method (Matreyek et al., 2020). All cells were split into fresh media and new flasks twice weekly. Stable hACE2‐HEK293Ts were cultured in complete DMEM supplemented with 2 μg/ml doxycycline in H_2_O. To transfect HEK293T cells to make SARS‐CoV‐2 spike‐lentivirus pseudotypes for the CCF2‐AM viral fusion assay, or to make EVs, cells were detached with 0.05% trypsin‐EDTA and 2.5 × 10^6^ cells re‐plated in 60.8 cm^2^ tissue culture in 10 ml of media. For making productive infection‐capable spike pseudotypes, 1.5 × 10^6^ cells were plated in a 6‐well tissue culture dish in 2 ml of media. For visualization of S‐EGFP expression in cells, 2.4 × 10^5^ cells were plated in a 35 mm glass‐bottom dish. For production of EVs for NFC, 1.2 × 10^6^ cells were plated in 6‐well tissue culture dishes in 3 ml of media. EV‐depleted DMEM was created by supplementing DMEM with 1% penicillin/streptomycin and 10% EV‐depleted FBS. EV‐depleted FBS was created by ultracentrifugation of HI‐FBS at 100,000 g for 16 h in a Beckman Coulter Optima L‐90K Ultracentrifuge using an SW32Ti rotor. EV‐depleted FBS was removed from the EV‐pellet, filtered at 0.22 μm, and stored at 4°C.

### 2.2 Plasmids

The core plasmid for making CCF2‐AM assay‐capable spike‐lentiviral vectors, pNL4‐3ΔEnvΔVpr‐EGFP, was derived from pNL4‐3ΔEnv‐EGFP, obtained from Drs. Haili Zhang, Yan Zhou, and Robert Siliciano and the AIDS Reagent Program (ARP). β‐Lactamase‐Vpr was generously provided by Dr. Robert Doms. The pCAGGS‐SARS‐CoV‐2 spike codon‐optimized plasmid was obtained from Dr. Paul Bates at the University of Pennsylvania. The pCAGGS‐SARS‐CoV‐2 D614G spike, used to make CCF2‐AM assay pseudovirus, was cloned from the pCAGGS‐SARS‐CoV‐2 spike plasmid using mutagenic primers and Gibson assembly. The pCAGGS‐SARS‐CoV‐2 spike‐EGFP plasmid was cloned from pCAGGS‐SARS‐CoV‐2 spike. The pCAGGS E, M and N plasmids were obtained from Dr. Paul Bates at the University of Pennsylvania. The pCAGGS N‐mRuby3 was cloned from pCAGGS N. Tetraspanin plasmids mEmerald‐CD9‐10 and mEmerald‐CD81‐10 were obtained from Addgene (gifts from Michael Davidson; Addgene plasmid #54029/54031) and used for cloning the CD9‐tagBFP2 and CD81‐mRuby3 constructs. The hACE2 plasmid was obtained Dr. Paul Bates at UPenn. The hACE2‐KanR plasmid was cloned by PCR and Gibson assembly. The core packaging plasmid for making productive infection spike lentivirus, psPAX2, was obtained from Addgene (gift from Didier Trono; Addgene plasmid #12260). The transfer plasmid for this pseudovirus, pLenti‐EGFP/mNeonGreen, was a kind gift from Dr. Kenneth Matreyek, derived from pLenti CMV rtTA3 Blast (w756‐1) (gift from Eric Campeau; Addgene plasmid #26429) by replacement of rtTA3 with EGFP‐P2A‐mNeonGreen and removal of EM7‐Blast. The codon optimized spike plasmid for this pseudovirus, G769B spike D614G, was a kind gift from Dr. Kenneth Matreyek.

### 2.3 Antibodies

The anti‐hACE2 Alexa Fluor 488 antibody used for flow cytometry evaluation of ACE2 expression was obtained from R&D Systems (Catalog no. FAB 9332G). The rabbit‐anti‐SARS‐CoV‐2 spike neutralizing antibody was obtained from Sino Biological (Catalog no. 40592‐R001). The normal rabbit IgG isotype control Ab was obtained from R&D Systems (Catalog no. AB‐105‐C). The calnexin antibody for western blot was obtained from Abcam (Catalog no. AB22595). The polyclonal anti‐spike (S2) antibody was obtained from Abcam (Catalog no. AB272504). The CD63 antibody was obtained from Abcam (Catalog no. AB59479).

### 2.4 Convalescent COVID‐19 patient sera

This study utilized convalescent serum samples collected from participants 45 to 110 days after symptomatic COVID‐19 infections, confirmed positive by PCR. Individuals signed informed consent for a protocol approved by University Hospitals of Cleveland and the New England IRB.

### 2.5 Quantitation of anti‐spike antibodies in patient sera

Full‐length S protein (S1/S2 furin cleavage site mutated – Acro Biosystems), and the receptor‐binding domain (RBD) (AA 319–541) of the S1 subunit, were conjugated to magnetic microbeads (Luminex) at 1 μg  antigen‐concentration to 0.5 ml of microbeads. Antigen‐specific IgG is detected in patient serum after mixing with Ag‐microbeads using PE‐conjugated Donkey F(ab)2 anti‐human IgG, with Fcγ (Jackson Immunological) added. Using Magpix assay system (BioRad, Inc), the mean fluorescent index is recorded. To provide an internal standard, a pool of convalescent plasma was generated from individuals with moderate to high antibodies to S and RBD following natural infection with virus collected more than 4 weeks after their COVID‐19 infection. Starting at a 1:100 dilution, the pool was titrated at half‐log dilutions of 1:500, 1:2500, 1:12,500 and 1:65,000 for a total of 5 dilutions, followed by MFI measurements to make six biological replicates. Using a log_10_ transformation of both dilutions and MFI values, a linear curve was generated with a slope of x, having an r^2^ consistently > 0.95 and a coefficient of variation < 10% across the replicates.  To generate relative antibody units (AU), the starting dilution of 1:100 was given the arbitrary value of 4000. AU decreased with increasing dilution out to 1:65,000, having an AU of 1.6.

### 2.6 S‐EGFP and N‐mRuby3 expression

To visualize cellular S‐EGFP expression, HEK293T cells were plated in 35 glass bottom dishes (Ibidi, Catalog no. 81158) plates as previously stated. The following day, 0.8 μg of S‐EGFP DNA and 0.4 μg each of pCAGGS E, pCAGGS M, and pCAGGS N‐mRuby3 were co‐transfected using polyethylenimine (PEI). 24 h after transfection, the cells were imaged using a DeltaVision Deconvolution Microscope with 100x magnification, 1040 × 1040 XY dimensions, and 1 × 1 binning. S‐EGFP was imaged using the FITC filter (475/28 excitation, 525/48 emission, 32% ND filter, 1.0 s exposure) and N‐mRuby3 was imaged using the TRITC filter (542/27 excitation, 597/45 emission, 50% ND filter, 0.10 s exposure). Images were deconvoluted and processed using the softWoRx software.

### 2.7 NFC of S‐EGFP EVs with E, M and N‐mRuby3 coexpression

Fluorescent EVs were produced by transfection of HEK293T cells 1 day after plating cells in 6‐well plates in EV‐depleted DMEM. 4 μg of S‐EGFP DNA and 2 μg each of pCAGGS E, pCAGGS M, and pCAGGS N‐mRuby3 were co‐transfected using polyethylenimine (PEI). Three days post‐transfection, EVs in the conditioned media were passed through a 0.45 μm filter to remove cell debris and immediately fixed in 4% PFA. Samples were diluted 1:200 into 0.1 μm filtered PBS. NFC experiments were immediately performed using a FACSAria II Special Order Research Project cell sorter (Becton Dickinson). A simultaneous threshold of 200 arbitrary units (determined based on mock EVs) was placed on all both fluorescent channels to allow the specific and exclusive detection of fluorescently‐labelled particles and VLPs.

### 2.8 Nanoscale flow cytometry (NFC) of S‐EGFP EVs

Fluorescent EVs were produced by transfection of HEK293T cells 1 day after plating cells in 6‐well plates in EV‐depleted DMEM. 5 μg of S‐EGFP DNA and either 2 μg of CD9‐tagBFP2 or 0.5 μg of CD81‐mRuby3 DNA were co‐transfected using polyethylenimine (PEI), after optimizing DNA concentrations to improve the resolution and detection of fluorescent EVs using NFC. Single‐fluorescent controls were made using each of the three fluorescent constructs. Additionally, some cells were mock‐transfected in order to make a negative control EV population to set up fluorescence thresholding. Three days post‐transfection, EVs in the conditioned media were passed through a 0.45 μm filter to remove cell debris and immediately fixed in 4% PFA. Samples were diluted 1:200 into 0.1 μm filtered PBS in order to minimize swarm detection, as described previously (Bonar & Tilton, 2017). NFC experiments were immediately performed using a FACSAria II Special Order Research Project cell sorter (Becton Dickinson). A simultaneous threshold of 200 arbitrary units (determined based on mock EVs) was placed on all three fluorescent channels to allow the specific and exclusive detection of fluorescently‐labelled particles. All samples were run with the same acquisition settings, including voltage, triggering threshold, and flow rate. More detail can be found in the supplemental MIFlowCyt‐EV Report.

### 2.9 Production of spike(+) EVs and spike(‐) EVs

HEK293T cells were plated in 60.8 cm^2^ tissue culture dishes as stated previously. The following day, each dish was transfected with 6 μg of pCAGGS SARS‐CoV‐2 spike plasmid (or mock transfected to make spike(‐) EVs) using JetOptimus. The media on the cells was replaced with fresh EV‐depleted DMEM 4 h post‐transfection. Three days post‐transfection, conditioned media from 12 dishes was passed through a 0.45 μm filter to remove cell debris, pooled, and EVs were concentrated and purified using tangential flow filtration (TFF).

### 2.10 Production of spike‐pseudotype lentivirus (S‐LVs) for CCF2‐AM entry assay

HEK293T cells were plated in 60.8 cm^2^ tissue culture dishes and the following day, each dish was transfected with 5 μg of pNL4‐3ΔEnvΔVpr‐EGFP HIV core plasmid, 3 μg of β‐lactamase‐Vpr plasmid, and 3 μg of pCAGGS SARS‐CoV‐2 D614G spike plasmid using JetOptimus. The media on the cells was replaced with fresh EV‐depleted DMEM 4 h post‐transfection. Three days post‐transfection, conditioned media from 6 dishes was passed through a 0.45 μm filter to remove cell debris, pooled, and LVs were concentrated and purified using tangential flow filtration (TFF).

### 2.11 Production of S‐LVs for productive infection assay

HEK293T cells were plated in 6‐well tissue culture dishes, and at the same time reverse‐transfected. Briefly, 0.6 μg each of psPAX2, pLenti‐EGFP/mNeonGreen, and G769B spike D614G were mixed in 80uL of DNA diluent (10 mM HEPES, 150 mM NaCl, H_2_O solution – 0.22 μm filter sterilized). 7 μg of PEI (1 μg/μl) was then added to reach an approximately 4:1 ratio of PEI to DNA. The DNA/PEI mixture was then added to the recently plated cells prior to attachment, after a 15 min room‐temperate incubation. The following day, after cells had attached, the media was replaced with 1 ml of EV‐depleted DMEM (D0). The viral supernatant was removed from each well, pooled, and replaced with fresh EV‐depleted DMEM every 24 h after until use in the productive infection assay on D3. For this assay, pooled viral supernatant was centrifuged at 300 g for 3 min to pellet cells and cell debris, and then transferred to a clean container before use.

### 2.12 Tangential flow filtration (TFF) – EV and LV concentration and purification

TFF is a filtration technique in which the sample is passed parallel to the filter, reducing filter clogging (‘fouling’) and improving purity and yields for large samples. We used 500 kDa MWCO TFF hollow‐fibre filters with a Repligen KrosFlo® KR2i TFF System that remove small contaminants while the larger EVs and viruses are retained and concentrated in the retentate. The purified EV and LV preps were washed with PBS to remove residual DMEM, concentrated to ∼2.5 mLs, and collected, aliquoted, and stored at ‐80°C until further use.

### 2.13 Mass spectrometry of spike(+) EVs

A total of 2.5 ml of TFF‐purified spike(+) EVs were ultracentrifuged at 110,000 *g* for 2 h using a SW55Ti rotor to pellet EVs. The EV pellet was lysed with 2% SDS mixed with protease inhibitor tablets (Roche Diagnostics). Sample were analysed by LC‐MS/MS using an Orbitrap Eclipse Tribrid mass spectrometer (Thermo Scientific) equipped with a nanoACQUITYTM Ultra‐high pressure liquid chromatography system (Waters). More details on analytical parameters and data analysis can be found in the supplemental methods.

### 2.14 Western blot of spike(‐) and spike(+) EVs

Cellular lysates were acquired by lysing mock‐transfected and spike‐transfected EV‐producer cells with RIPA buffer after removal of the EV conditioned media. TFF‐purified spike(‐) and spike(+) EVs and corresponding cell lysates were heated at 37°C (calnexin blot and CD63 blot) or boiled at 95°C (spike blot). After equalizing RIPA and PBS content between lysates and EVs, samples were loaded and run on SDS‐PAGE gels (CD63‐blot was run under non‐reducing conditions). For the Calnexin and SARS‐CoV‐2 spike blot, 1 μl of lysate and 15 μl of EVs were loaded. For the CD63 blot, 1 μl of lysate and 1 μl of EVs were loaded. Blots were then probed for calnexin, SARS‐CoV‐2 spike, or CD63 at 1:1000 Ab dilutions.

### 2.15 Electron microscopy

Spike(‐) and spike(+) EV‐conditioned media from 60.8 cm^2^ tissue culture dishes was collected and 0.45 μm filtered to remove cells and large cell debris. Conditioned media was then overlaid onto a 30% sucrose/D_2_O/Tris (200 mM) cushion in ultracentrifuge tubes (Théry et al., 2006). EVs were ultracentrifuged at 100,00 g for 75 min using a SW32Ti rotor to remove small cell debris. The sucrose/D_2_O layer, containing EVs, was then removed, pooled, washed with PBS, and purified and concentrated to 1 ml using TFF. Next, 1 ml of TFF‐purified EVs was fixed in 4% paraformaldehyde. EVs were then deposited onto Formvar‐carbon coated EM grids and washed with PBS before transfer to 1% glutaraldehyde for additional fixation. After several washes with water, EVs were exposed to uranyl oxalate solution (pH 7) to improve the contrast of particles. EVs were then embedded in a mixture of uranyl acetate and 2% methyl cellulose. Sample grids were dried, then observed and imaged under the FEI Tecnai G2 Spirit BioTWIN with a Orius 832 CCD Camera at 80 kV.

### 2.16 Microfluidic resistive pulse sensing (MRPS)

MRPS is a microchip‐based technique that measures the concentration and size of EVs and other nanoparticles. Briefly, nanoparticles pass through a pore across which an electrical current is run; as particles pass through and obstruct the pore, the electrical resistance is altered in a manner proportional to the volume of the particle. Using a Spectradyne nCS1™ MRPS instrument, and C‐400 cartridges with a measurable size range of 65–400 nm, we analysed 3 μl of every TFF‐purified EV or LV sample. Approximately 20,000 events were collected, and then size and concentration data were compiled and exported for analysis. MRPS was used to determine the concentration of EV and LV samples for subsequent infection and neutralization experiments.

### 2.17 ACE2 transient transfection

To make HEK293Ts that transiently express ACE2, HEK293Ts were plated in 60.8 cm^2^ tissue culture dishes as stated previously. The following day, each dish was transfected with 8 μg of hACE2‐KanR plasmid using JetOptimus. The media on the cells was replaced with fresh media 4 h post‐transfection. The cells were detached using trypsin, counted, and re‐plated for use in the LV entry assay 48 h post‐transfection.

### 2.18 ACE2‐expression analysis

Forty‐eight hours post‐transfection, two 60.8 cm^2^ tissue culture dishes of ACE2‐transfected HEK293T cells were detached using trypsin to obtain a single cell suspension. After pooling and counting, one million ACE2‐transfected HEK293T cells were stained with 1 μl of anti‐ACE2‐FITC Ab for 30 min at 25°C. Cells were fixed in 1% paraformaldehyde and analysed via flow cytometry using a BD LSRII cytometer. ACE2 expression was analysed using the 488 nm laser.

### 2.19 S‐LV entry and neutralization assay

Prior to addition to cells, S‐LVs were thawed and 150 μl of S‐LVs were distributed to a 1.5 ml Eppendorf tube for each experimental condition. The number of total particles in the 150 μl S‐LV sample was determined using MRPS and that number used to determine the volume of spike(+) EVs or spike(‐) EVs to add to reach 1:1 or 1:2 S‐LV:EV ratios. For the 1:0 ratio, no EVs were added. After vortexing to mix well, nAb (or PBS for the 0 μg/ml condition) was added at the desired concentrations for all samples. For controls, an isotype‐matched antibody was used instead of the nAb. After vortexing again, the S‐LV/EV/nAb mixture was incubated at 4°C for 1 h to allow neutralization to occur. After 1 h, volumes were equalized to 495 μl between all conditions using 0.1 μm‐filtered PBS.

During the LV neutralization step, ACE2‐HEK293T cells were plated in 96‐well v‐bottom plates at 1.0 × 10^5^ cells/well. A total of 165 μls of the S‐LV/EV/nAb mixture were then added to each well, allowing each experimental condition to be performed with three technical replicates (PBS was added to some wells to establish assay background). The nAb experiments were also performed in three biological replicates using different preparations of S‐LVs and EVs on different days. Cells were spinoculated at 1200 × *g* and 25°C for 2 h and then incubated at 37°C for 1 h in a tissue culture incubator. Target cells were loaded with 2 μM CCF2‐AM dye, using the Invitrogen LiveBLAzer™ FRET‐B/G loading kit. Cells were incubated in CO_2_‐independent media (ThermoFisher) containing 2.5 mM probenecid at 25°C overnight to prevent cells from exporting cytosolic CCF2 dye. The following day (18 h following addition of probenecid), target cells were washed and stained with Invitrogen LIVE/DEAD™ fixable yellow stain for 30 min at 4°C. Finally, cells were washed again and fixed with 1% paraformaldehyde for flow cytometry analysis. Fusion data were collected by assessing EV‐mediated cleavage of cellular CCF2 FRET dye, using a BD LSRFortessa analytical flow cytometer. Approximately 15,000 events were collected per condition. All flow cytometry data were analysed in FlowJo (Treestar).

### 2.20 S‐LV productive infection and neutralization assay

Serum S‐LV productive infection and neutralization assays were performed in 96‐well flat‐bottom tissue culture dishes. Convalescent COVID‐19 patient serum was thawed, and diluted with PBS as necessary to obtain final dilutions of 1:50, 1:100, 1:150, and 1:200 after addition of all assay components. Each well received 50 μl of diluted serum, 50 μl (2.45 × 10^10^ EVs) of spike(‐) or spike(+) EVs, 50 μl of S‐LV viral supernatant from the productive infection S‐LV protocol, and 50 μl (10,000 cells) of stable hACE2‐HEK293Ts in Doxycycline‐DMEM. Each serum dilution was assayed in technical triplicate under each EV condition. An infection control was made by adding together only cells and S‐LV supernatant, replacing the EV and serum components with an equal volume of PBS. Cells were then incubated at 37°C in a tissue culture incubator. Approximately 72‐h post infection, cells were trypsinized and then fixed in 230 μl 1% paraformaldehyde. Infection data were collected using an Attune NxT analytical flow cytometer. Productive infection was assessed by determining the percentage of miRFP670+(ACE2+) EGFP/mNeonGreen+ cells within all miRFP670+ cells. Infection levels were then normalized to the no serum/no EVs infection control. The average number of cells examined was approximately 37,000. All flow cytometry data were analysed in FlowJo (Treestar).

### 2.21 Statistics

Unless otherwise noted, all figures display the average of three independent biological experiments, with error bars representing standard deviation. Where applicable, each point is the average of three *technical* replicates within an independent experiment. Statistical significance of the data in Figure 3b was tested by one‐way ANOVA with Dunnett's multiple comparisons test. In Figure 4c, significance was tested by ordinary two‐way ANOVA with Sidak's multiple comparisons test. In Figure 5a and Supplemental Figure 4, significance was tested by multiple T‐tests with Holm‐Sidak correction for multiple comparisons. In Figure 5c, significance was tested by one‐way ANOVA with Holm‐Sidak correction for multiple comparisons. Any p value less than 0.05 was considered statistically significant (*P*  > 0.05 = ns, not significant; or ns†, significant prior to correction for multiple comparisons; *P* ≤ 0.05 = *; *P* ≤ 0.01 = **; *P* ≤ 0.001 = ***; *P* ≤ 0.0001 = ****). For all statistical analyses and curve fits, we used GraphPad Prism 7. Curve fits and IC50 interpolations were performed with the [inhibitor] versus response (three parameters) nonlinear regression fit function . The correlations in Figure 5b and Supplemental Figure 3 were performed using the Pearson correlation function, reporting a two‐tailed *P*‐value. This correlation was visualized by overlaying a semi‐log or linear regression line on the plot. Percent neutralization in S‐LV entry assays was calculated as follows: $100-[(\frac{\%Entry-background}{\%Entry_{0\;nAb}-background})\times \;100]$. Percent of max infection in S‐LV productive infection assays was calculated as follows: $(\frac{\%infection(+sera+EVs)}{\%infection(-sera-EVs)})\times \;100$. No background subtraction was necessary in productive infection assays. EV decoy effect was calculated as follows:

(1) $$\mathrm{normalized}\;\mathrm{mean}\;\mathrm{infection}\left(\mathrm{spike}(+)\mathrm{EVs}\right)-\mathrm{normalized}\;\mathrm{mean}\;\mathrm{infection}\left(\mathrm{spike}(-)\mathrm{EVs}\right)$$

## 3 RESULTS

### 3.1 Expression of a tagged SARS‐CoV‐2 spike (S) protein in vitro

**FIGURE 1:**
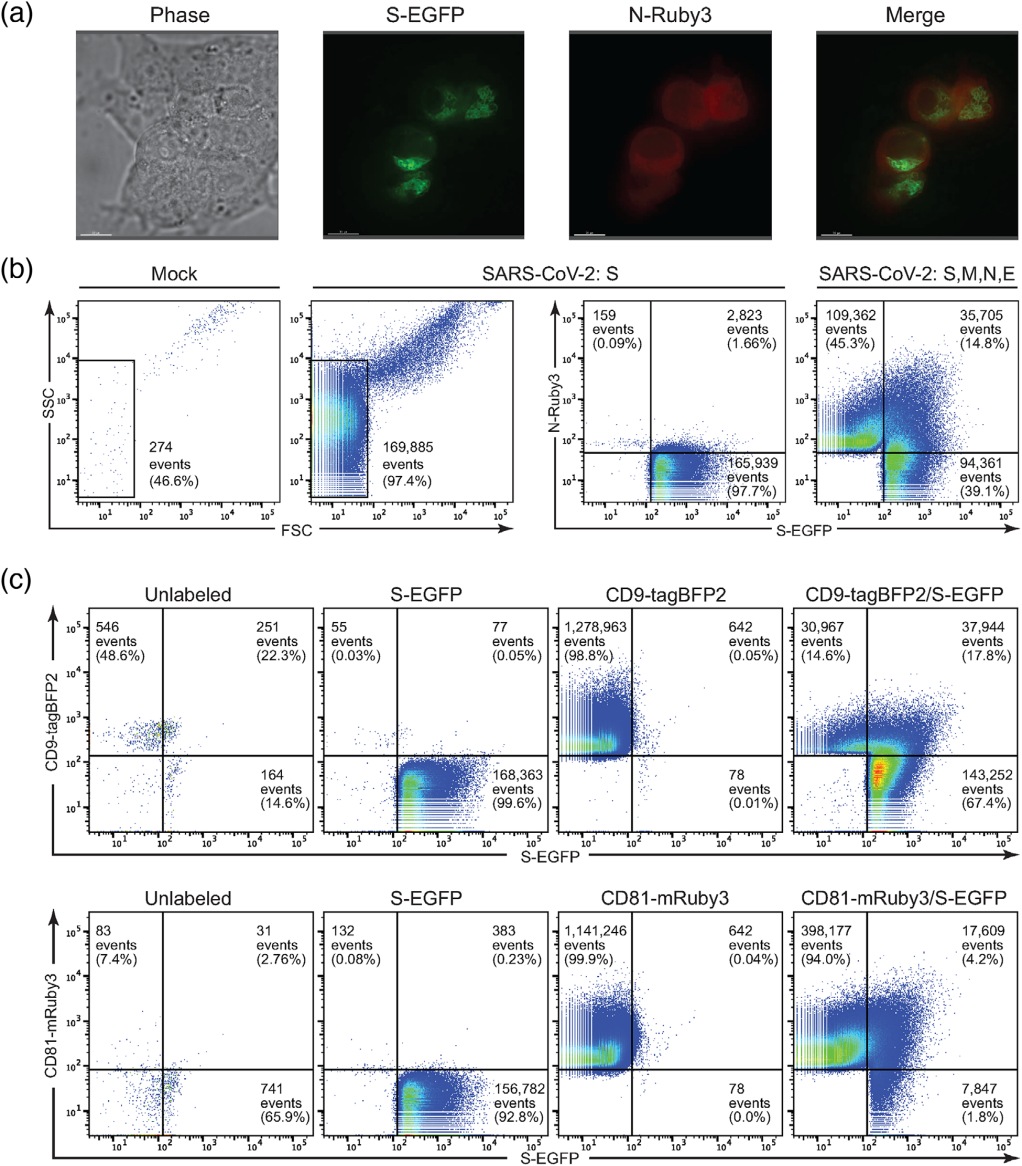
An EGFP‐tagged SARS‐CoV‐2 spike (S‐EGFP) protein is expressed well in vitro and is incorporated into particles bearing markers of extracellular vesicles. (a) Fluorescent and phase images of HEK293T cells transiently transfected with SARS‐CoV‐2 S‐EGFP, E, M and N‐mRuby3. S‐EGFP is readily detected and displays a perinuclear localization, while N‐mRuby3 had more diffuse expression. (b) Nanoscale flow cytometry (NFC) plots comparing EGFP+ particles in the supernatant of mock, S‐EGFP, and S‐EGFP/E/M/N‐mRuby3‐transfected HEK293Ts. Transfection with S‐EGFP resulted in a > 600‐fold increase in FSC^low^ events over mock transfection. S‐EGFP+ particles were readily detectible under S‐EGFP and S‐EGFP/E/M/N‐mRuby3 transfection conditions. Coexpression revealed a population of S‐EGFP+ N‐mRuby3+ events, likely VLPs. (c) NFC plots of particles in the supernatant of HEK293T cells transfected with S‐EGFP, CD9‐tagBFP2 and CD81‐mRuby3 plasmids. The 'unlabelled’ panels represent supernatant particles from mock‐transfected cells. The ‘S‐EGFP’, ‘CD9‐tagBFP2’, and ‘CD81‐mRuby3’ panels are supernatant particles from HEK293T cells transfected with each construct individually, to determine channel spillover and draw gates. The rightmost panels consist of particles from cells co‐expressing S‐EGFP with either CD9‐tagBFP2 or CD81‐mRuby3

To monitor expression of the SARS‐CoV‐2 spike protein within cells, we generated fluorescently tagged versions of spike (S‐EGFP) and transfected them into HEK293T as well as A549 and HBEC3‐KT lung epithelial cells. HEK293T cells were readily transfected and S‐EGFP demonstrated a perinuclear localization (data not shown), consistent with its reported ERGIC localization (Duan et al., 2020). In contrast, A549 and HBEC3‐KT were far more resistant to transfection with a variety of reagents. In a few cases, fluorescence was observed after 16—24 h but was rapidly followed by rounding and detachment of cells, suggesting that the spike protein could be cytotoxic in absence of other viral factors, as has previously been reported. We next co‐transfected S‐EGFP and the viral Membrane (M), Envelope (E), and Nucleocapsid (N) proteins, the latter linked to mRuby3 (N‐Ruby3). Again, HEK293T cells were readily transfected without evidence of cytotoxic effects, and showed ERGIC localization of spike (Figure 1a). In contrast to the perinuclear distribution of spike, nucleocapsid demonstrated much more diffuse cytoplasmic staining. Unfortunately, the lung cell lines remained difficult to transfect and sensitive to SARS‐CoV‐2 protein expression.

We continued by characterizing SARS‐CoV‐2 proteins expression in extracellular vesicles (EVs) from HEK293T cells, which are widely used in viral research. Although detection of extremely small particles such as viruses or EVs by light scatter alone using nanoscale flow cytometry (NFC) is challenging, NFC readily detects particles that are labelled with fluorescent proteins (Bonar & Tilton, 2017; Morales‐Kastresana et al., 2017; Pasalic et al., 2016; Welsh et al., 2020). The flow cytometer detected a low number of particles in the supernatants of unlabelled, mock‐transfected cells (Figure 1b) and many of these were SSC^high^FSC^high^ events we have determined to be aggregates in NFC of viruses (Bonar & Tilton, 2017). Using fluorescence to detect particles, we observed an over 600‐fold increase in FSC^low^ events in the supernatant from S‐EGFP transfected cells compared to mock‐transfected cells. In the cells co‐expressing S‐EGFP, E, M, and N‐Ruby3, we observed a large number of particles expressing S‐EGFP (39.1%) or N‐Ruby3 (45.3%) only and a smaller population co‐expressing both labelled proteins (14.8%). The population of vesicles containing both viral S and N proteins likely represent virus‐like particles (VLPs) – viruses lacking their genomes and key protein components. The vesicles containing N protein alone would be considered ‘defective viral particles’ from a virology perspective, consisting of viral structural components that failed to recruit spike and are non‐infectious. Finally, we hypothesized that the population of vesicles containing S‐EGFP alone were likely EVs that have incorporated spike protein. Since EVs are similar in size and density to viruses and VLPs, it was not experimentally practical to co‐transfect S, M, N, and E proteins and then purify or isolate the S‐containing EVs away from VLPs or defective viral particles. For these reasons, along with good expression and lack of toxicity, we expressed spike protein in isolation to better characterize its ability to be incorporated into EVs.

To test that S protein was being incorporated into EVs, we expressed S‐EGFP and a fluorescently‐tagged version of two EV markers, tetraspanins CD9‐tagBFP2 or CD81‐mRuby3. In our CD9 co‐expression experiment, NFC analysis demonstrated that approximately 85% of detected particles carried S‐EGFP (Figure 1c, upper panels). Approximately 20% of spike+ EVs also expressed CD9+, while ∼80% were CD9‐, suggesting that S‐EGFP can be recruited into different subsets of EVs as classified by tetraspanin incorporation. HEK‐derived CD9+ EVs, with or without S‐EGFP, are relatively rare. Supporting this idea, the CD81 co‐expression experiment showed approximately 6.0% of detected particles carrying S‐EGFP; nearly 70% of which were CD81+ (Figure 1c, lower panels). Overall, CD81+ EVs, whether carrying S‐EGFP or not, were much more prevalent than CD9+ EVs, and likely represent a large fraction of the detectable S‐EGFP+ population. The majority of the detected CD81+ EVs were CD81+ and S‐EGFP‐, which could be due to lower levels of S‐EGFP incorporation compared to CD81 or, alternatively, could indicate that S‐EGFP may only be present on a fraction of CD81+ EVs. Regardless, the NFC data clearly indicate populations of EVs that incorporate both S‐EGFP and EV markers CD9 or CD81. Taken together, these data demonstrate that fluorescently‐labelled SARS‐CoV‐2 spike protein can be efficiently expressed in HEK293T cells and is incorporated into shed particles that bear markers of extracellular vesicles.

### 3.2 SARS‐CoV‐2 spike protein is incorporated into extracellular vesicles (EVs)

**FIGURE 2:**
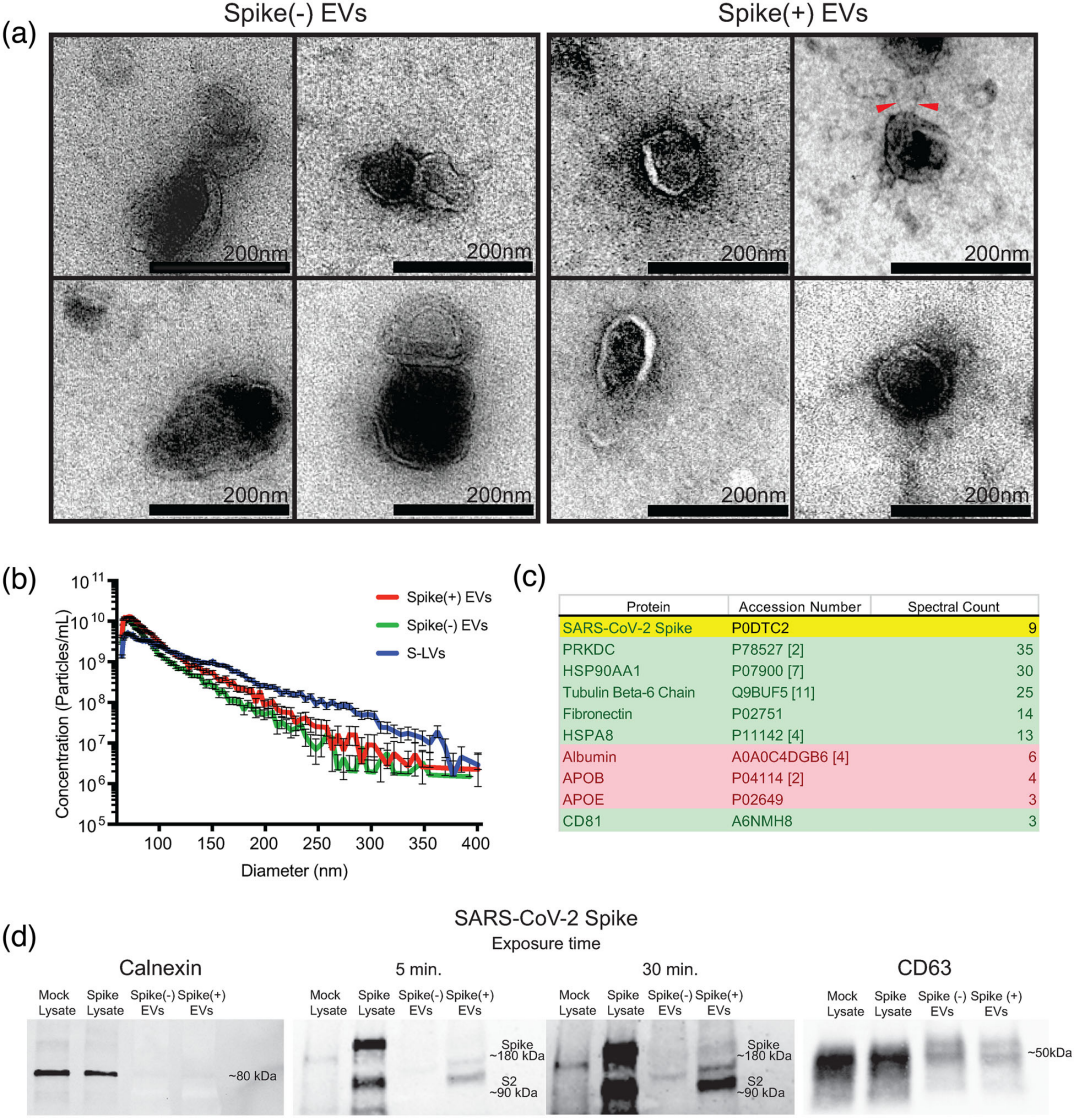
The wild‐type SARS‐CoV‐2 spike is incorporated into extracellular vesicles. (a) Transmission electron micrographs of purified extracellular vesicles produced by mock‐transfected (left box) or SARS‐CoV‐2 spike‐transfected (right box) HEK293Ts. The scale bar for all of the inset images is 200 nm. Red arrowheads point to stalk like protrusions in spike(+) EVs. The ‘cup‐shaped’ morphologies of collapsed vesicles are visible in the images. (b) Size distribution plot created using microfluidic resistive pulse sensing (MRPS) data of spike(+) and spike(‐) EVs, and S‐LVs. The microfluidic cartridges utilized for these experiments measure particles 65–400 nm diameter. (c) Mass spectrometry analysis of purified EVs. Mass spectrometry revealed the presence of SARS‐CoV‐2 spike protein in the purified EV population. Traditional EV‐associated proteins are highlighted in green and common EV‐preparation contaminants are highlighted in red. The ‘spectral count’ refers to the number of unique peptide fragments detected and identified as belonging to the listed protein and is commonly used as a surrogate measure of protein abundance. The full list of identified proteins can be found in the supplemental files. (d) Western blots of mock and spike‐transfected cell lysates, and spike(‐) and spike(+) EVs. Calnexin is visible in cell lysates as a ∼80 kDa band. Full length SARS‐CoV‐2 spike is visible as a ∼180 kDa band, and S2 subunit is visible as a ∼90 kDa band. The anti‐spike antibody did lead to some non‐specific banding in mock‐transfected controls. CD63 is visible as a smear band at ∼50 kDa

To determine whether the untagged, wild‐type SARS‐CoV‐2 spike protein can also be incorporated into EVs, we transfected HEK293T cells with a plasmid expressing full‐length, codon‐optimized spike protein and followed MISEV 2018 guidelines for EV production, concentration, and characterization. As characterization of extracellular vesicles is important in research proposing that they have a biological effect, we assessed the EVs using electron microscopy, microfluidic resistive pulse sensing (MRPS), mass spectrometry, and western blot. Transmission electron microscopy on negative stained EVs harvested and purified from mock‐ and spike‐transfected cells revealed vesicles that were intact, approximately 50–250 nm in diameter, and with a distinct double membrane (Figure 2a). The “cup‐shaped” structure of vesicles was also visible, a morphology often seen with EM images of EVs that is caused by the negative staining preparation. We did not observe the characteristic electron microscopic ‘halo’ of spike proteins, for which the coronavirus family is named. However, we did occasionally observe EVs with distinct linear protrusions from the membranes; these were exclusively seen in the spike(+) EV samples. Next, we analysed the total size distribution and concentration of our EV preps using MRPS, which excludes larger particles and aggregates with Next, we analysed the total size distribution and concentration of our EV preps using MRPS, which excludes larger particles and aggregates with a microfluidic chamber to and then measures electrical disturbances caused by nanoparticles passing through a small pore. MRPS revealed a distribution of EVs ranging from 65–400 nm in diameter, with the majority of EVs being on the smaller end of the spectrum (Figure 2b). There was no notable difference in concentration or size distribution between spike (+) and spike (‐) EVs. For comparison, supernatants containing spike‐pseudotyped lentiviral particles (S‐LVs) tended to be slightly larger in size distribution.

An unbiased mass spectrometry analysis of purified spike(+) EVs identified multiple proteins commonly found enriched in EV proteomics data sets, including high levels of PRKDC, HSP90AA1, and HSPA8 (Figure 2c). Levels of common EV contaminants, such as albumin and apolipoproteins, were comparatively low. In addition, mass spectrometry detected five peptides originating from SARS‐CoV‐2 spike protein. One of these peptides originated from the ACE2 receptor‐binding domain (RBD) of the S1 subunit, while the other four came from S2, suggesting that EVs can contain full‐length, wild‐type spike protein in addition to the tagged S‐EGFP. To clarify this further, we investigated the conformation of spike on EVs and cell lysates by western blotting. The ER chaperone calnexin, which is excluded from EVs, was detected in mock‐ and spike‐transfected HEK293T cell lysates but not EV preparations (Figure 2d). In contrast, the EV‐associated tetraspanin CD63 was expressed at high levels in both lysates and EV preparations. Using an antibody directed at the C‐terminus of spike antibody, we determined that spike‐transfected cell lysates expressed both full‐length spike and the S2 subunit that is formed when the S1 domain is lost. Compared to the cell lysates, the majority of EV‐borne spike appeared to have shed the S1 subunit, although a band for full‐length spike was observable with longer exposure. Together, these data indicate that the SARS‐CoV‐2 spike protein is incorporated into EVs, predominantly as the S2 subunit with rarer full‐length glycoproteins.

### 3.3 A SARS‐CoV‐2 infection model that is inhibited by neutralizing antibodies

Lentiviruses can incorporate the spike or envelope proteins from a variety of different viruses. These hybrid viruses, called *pseudoviruses*, are used to safely study the entry mechanisms of pathogenic viruses including HIV, Ebola, and SARS‐CoV‐2 (Li et al., 2018; Nie et al., 2020; Salata et al., 2019). Here, we produced lentivirus (LVs) pseudotyped with SARS‐CoV‐2 spike protein (S‐LVs), specifically the D614G version of spike that has been reported to improve pseudotyping efficiency and increase infectivity (Korber et al., 2020; Maurano et al., 2020; Zhang et al., 2020). The D614G spike mutant emerged as the SARS‐CoV‐2 pandemic spread through Europe and subsequently dominated the outbreaks in North and South America (Korber et al., 2020; Maurano et al., 2020). While the rapid spread of the D614G mutant in Europe and the Americas indicate that the mutation does convey a fitness advantage, its susceptibility to antibody neutralization remains similar or higher to that of the wild‐type spike (Weissman et al., 2020; Zhang et al., 2020). S‐LVs were produced by transiently transfecting HEK293T cells with plasmids encoding lentiviral structural proteins, SARS‐CoV‐2 spike, and a β‐lactamase–Vpr protein that is specifically incorporated into LV particles. If the particles undergo spike‐dependent fusion with target cells, they release β‐lactamase into the cytoplasm that cleaves a fluorescent substrate and causes a measurable fluorescent change in the cells. This assay has been widely used in viral research (Jones & Padilla‐Parra, 2016).

**FIGURE 3:**
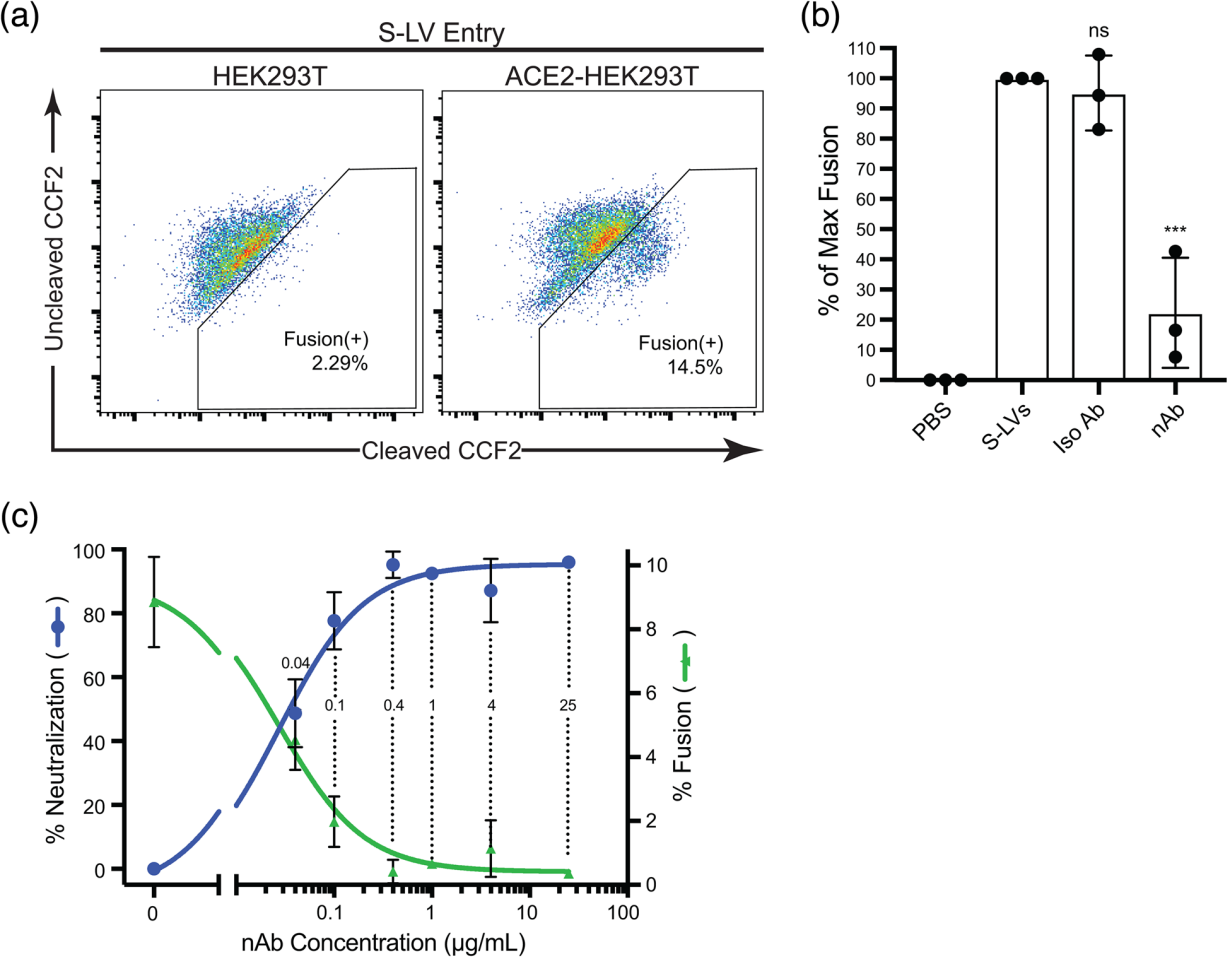
S‐LV entry and neutralization in ACE2‐HEK293Ts. (a) Representative flow cytometry plots of S‐LV entry into HEK293T cells demonstrating dependence on ACE2 expression. The left panel shows S‐LV entry into untransfected HEK293Ts, with a small amount of background dye conversion (also appearing in the uninfected control) appearing in the fusion+ gate. The right panel shows S‐LV entry into HEK293Ts transiently transfected with human ACE2. The Y‐axis represents the uncleaved CCF2 dye signal, while the X‐axis represents CCF2 that has been cleaved by β‐lactamase. (b) S‐LV entry assay in the presence of 25 μg/ml anti‐spike nAb or isotype control antibody. Entry in the absence of nAb was normalized to 100%. Significance was measured by one‐way ANOVA followed by Dunnett's multiple comparisons test. Data are displayed as mean + SD of three independent biological experiments (n = 3) and each point within a column is the mean of three technical replicates. ***, *P* ≤ 0.001. (c) S‐LV fusion at differing nAb concentrations and neutralization dose curve. The right Y‐axis represents S‐LV entry into ACE2‐HEK293Ts with the background subtracted; the left Y‐axis presents percent neutralization of S‐LV entry, which is calculated from the fusion data as described in the methods. Data are displayed as mean + SD of three technical replicates. Numbers within dotted lines refer to nAb concentrations tested

First, we transfected HEK293Ts with ACE2 plasmid and confirmed expression in a large fraction of cells via flow cytometry (Supplemental Figure 1). We then assessed the ability of S‐LVs to infect HEK293T cells, with or without transient ACE2 expression. S‐LVs entry into HEK293Ts was dependent upon transient expression of exogenous ACE2, as expected for SARS‐CoV‐2 spike‐mediated entry (Figure 3a). S‐LV entry into ACE2‐expressing HEK293T cells was significantly higher than the background (determined by adding PBS to cells) of our assay with all S‐LV preparations. Since independent preparations of LVs often vary in their efficiency of viral entry, we normalized by setting lentiviral entry in the absence of neutralizing antibodies to 100% and assessed the ability of anti‐spike neutralizing antibody to block S‐LV entry. At the manufacturer‐recommended concentration of 25 μg/ml, this nAb significantly neutralized S‐LV entry into ACE2‐293Ts (*P* = 0.0006), while its corresponding isotype control antibody did not (*P* = 0.8572) (Figure 3b).

To further characterize the S‐LVs and their ability to be neutralized by antibody, we generated a neutralization dose‐curve. We tested a range of different nAb concentrations within our ACE2‐293T system and found that concentrations as low as 0.4 μg/ml resulted in nearly complete neutralization of S‐LV entry (Figure 3c, blue line). Other concentrations, such as 0.04 μg/ml and 0.1 μg/ml, were close to IC_50_ and IC_80_, respectively. Moving forward, we decided to use two concentrations near IC_100_ (1 μg/ml and 0.4 μg/ml), and the two below IC_100_ (0.04 μg/ml and 0.1 μg/ml), in addition to our 0 μg/ml nAb condition, in order to build nAb neutralization curves and monitor changes in nAb efficiency. These data demonstrate that we have a working LV pseudotype entry assay to use as a surrogate of SARS‐CoV‐2 infection that shows expected characteristics of spike‐mediated viral entry: a dependence on ACE2 expression and an ability to be neutralized by spike‐specific nAbs.

### 3.4 EVs bearing SARS‐CoV‐2 spike protein inhibit commercial antibody‐mediated neutralization of viruses

Our NFC and proteomics data clearly demonstrated that full length and truncated spike protein was present on EVs, but it remained unclear whether it was properly folded on the EV surface. We reasoned that EV‐borne spike, if present in its native conformation, could potentially bind to nAbs and reduce the effective concentration of ‘free’ nAbs available to bind and inhibit viruses. This could enable spike(+) EVs to serve as immune decoys in a manner similar to the defective and sub‐viral particles released by HIV and HBV, respectively, that bind nAbs and reduce their efficiency.

**FIGURE 4:**
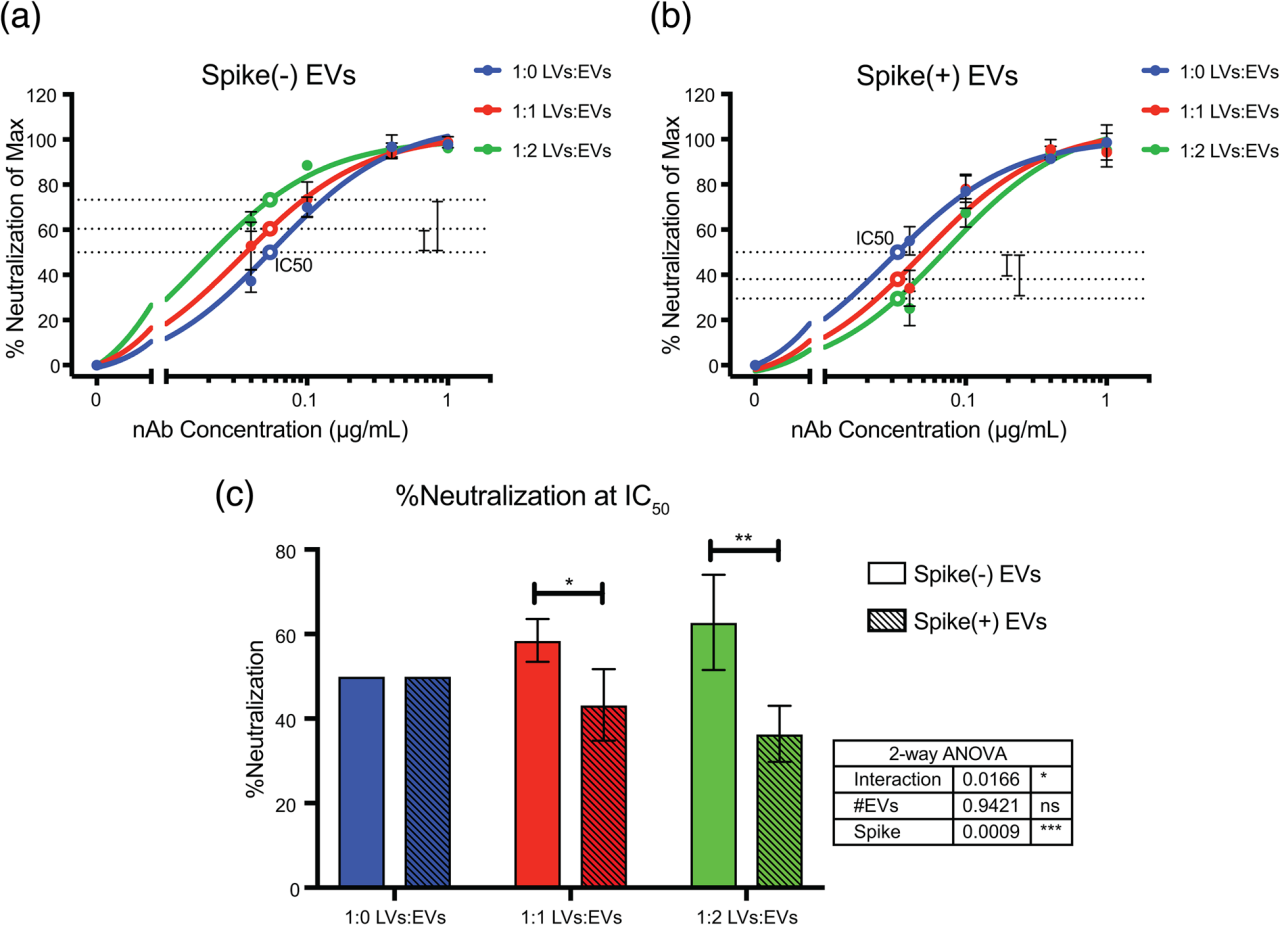
Spike(+) EVs reduce the efficiency of commercial neutralizing antibodies at blocking SARS‐CoV‐2 spike‐dependent viral entry. Representative dose curves of nAb‐mediated inhibition of S‐LV entry in the presence of spike(‐) EVs (a) and spike(+) EVs (b) at varying ratios. Data are displayed as mean ± SD of technical triplicates. The open point on the blue line (1:0 LVs:EVs) represents the interpolated IC_50_ value. The open points on the red and green lines represent the interpolated % neutralization value at the IC_50_ concentration determined from the blue line. Dashed lines are a visual representation of the changes in neutralization efficiency induced by spike(‐) or (+) EVs. (c) Summary data from three independent biological replicates of nAb efficacy in the presence of spike(‐) and spike(+) EVs. Data are displayed as mean + SD of three independent biological (n = 3) experiments. Solid filled columns are spike(‐) EVs and dashed fill columns are spike(+) EVs. Significance was measured by two‐way ANOVA followed by Sidak's multiple comparisons test. Inset table displays ANOVA p‐values and contributions of variables to overall variance. Asterisks over the columns represent the *P*‐values of the multiple comparisons tests. *, *P* ≤ 0.05; **, *P* ≤ 0.01; ***, *P* ≤ 0.001

To study the effects of spike(+) EVs and spike(‐) EVs, we added them at 1:0, 1:1, and 1:2 particle count‐ratios relative to S‐LVs and measured changes in nAb efficacy. To quantify these changes, we built nAb neutralization curves and then interpolated and compared the amount of neutralization with spike(‐) (Figure 4a and Supplemental Figure 2A) and spike(+) (Figure 4b and Supplemental Figure 2B) EVs at the antibody concentrations that neutralized 50% of virus (IC50) in the absence of EVs. At the 1:1 LV:EV ratio, spike(‐) EVs unexpectedly enhanced predicted neutralization efficiency of the nAbs to an average of 58.51% ± 5.09%, whereas spike(+) EVs reduced LV neutralization down to an average of 43.24% ± 8.45%. The difference in predicted neutralization efficiency with the spike(+) and (‐) EVs was significant (*P* = 0.0475) (Figure 4c), demonstrating that the presence of spike on EVs affects the efficiency of nAbs. To our knowledge, this spike(‐) EV‐mediated enhancement of Ab activity has not previously been reported. At the 1:2 LV:EV ratio, spike(‐) EVs enhanced predicted neutralization to 62.74% ± 11.26% while spike(+) EVs decreased neutralization even further, down to an average of 36.39% ± 6.62% (*P* = 0.0012)(Figure 4c). These data demonstrate a dose‐dependent, inhibitory effect of spike(+) EVs on antibody‐mediated neutralization of viruses bearing the SARS‐CoV‐2 spike protein.

While only a small fraction of the EVs contain spike, it is clear that this population is capable of having an effect on nAb efficiency. Given that the spike(‐) EVs enhanced predicted nAb efficiency, the inhibitory effect caused by this small proportion of spike(+) EVs seems to be quite outsized. Taken together, these data suggest that SARS‐CoV‐2 spike on the surface of EVs can serve as decoys for a commercial neutralizing antibody, reducing its effectiveness in preventing spike‐mediate viral entry.

### 3.5 EVs bearing SARS‐CoV‐2 spike protein inhibit virus neutralization by convalescent patient serum

While commercial monoclonal nAbs are a useful tool for investigating viral neutralization, they are not necessarily representative of the more complex biological interactions that occur in the human body. Human serum can contain a variety of different neutralizing antibodies targeting spike epitopes, as well as non‐neutralizing antibodies that contribute to other forms of antiviral responses. In order to assess whether our spike(+) EVs could retain their decoy effects in a more biologically‐relevant context, we obtained serum from nine convalescent COVID‐19 patients. Given the need for larger scale experiments, we used a different spike‐lentiviral (S‐LV) pseudotype system that produced D614G S‐LVs capable of infecting ACE2‐293T cells and producing EGFP and mNeonGreen upon productive infection.

**FIGURE 5:**
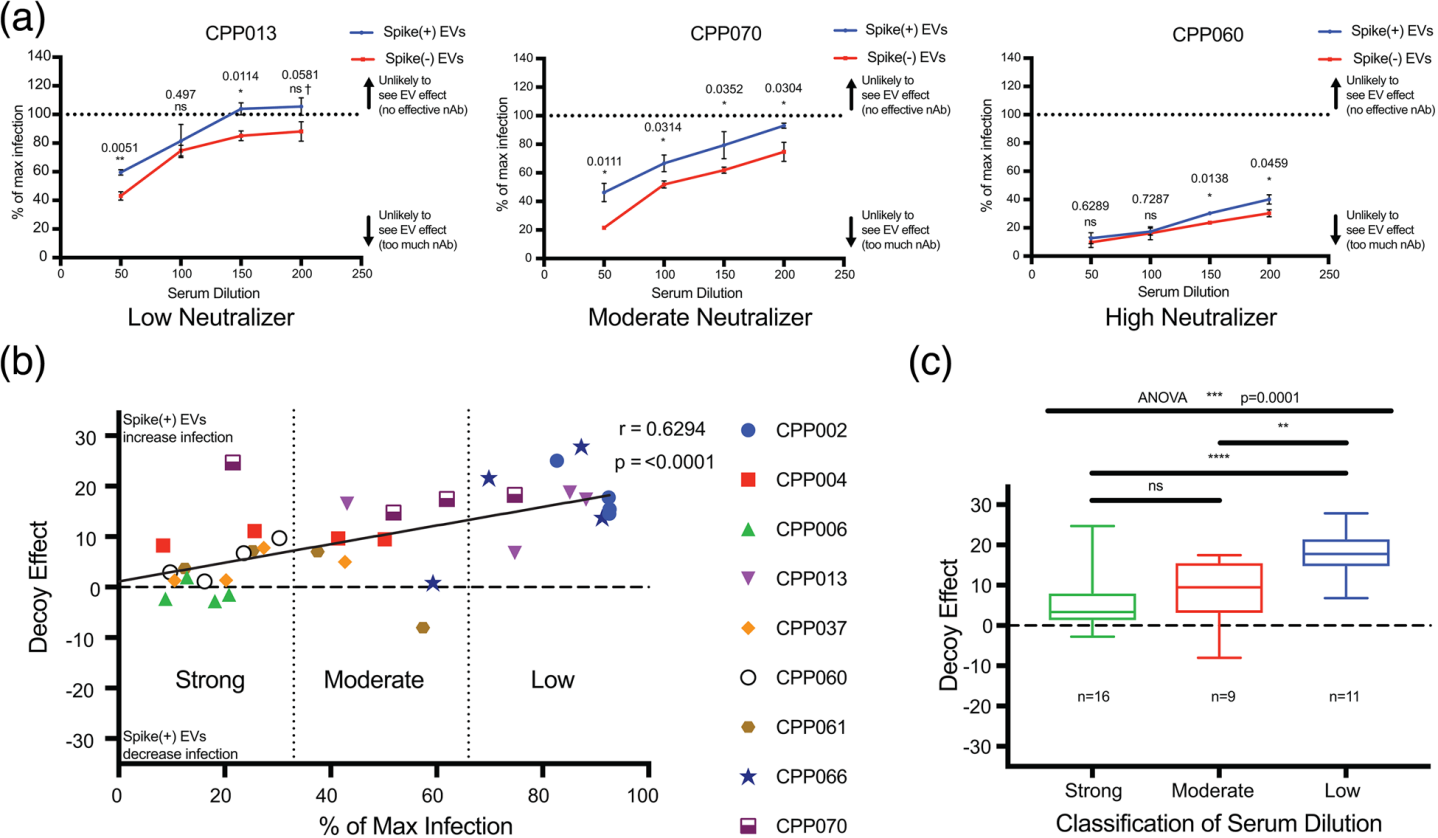
Spike(+) EVs reduce the neutralizing ability of convalescent COVID‐19 patient sera. (a) COVID‐19 convalescent serum S‐LV neutralization curves from three different patients representing low, moderate and high neutralization ability. Curves represent serum neutralization in the presence of spike(‐) EVs (red) and spike(+) EVs (blue). The Y‐axis represents S‐LV infection levels normalized to a no serum/no EVs infection control (termed max infection) a calculation described in the methods. The X‐axis represents fold‐dilution of the patient serum. Data are displayed as mean ± SD of technical triplicates. Significance was measured by multiple T‐tests followed by Holm‐Sidak correction for multiple comparisons. Asterisks over the columns represent the *P*‐values of the T‐tests after correction for multiple comparisons. ns, not significant; ns†, significant prior to correction for multiple comparisons; *, *P* ≤ 0.05; **, *P* ≤ 0.01. (b) Aggregated COVID‐19 convalescent serum neutralization data from nine patients. Scatter plot showing the relationship between the EV decoy effect and neutralization ability of serum. The Y‐axis represents EV decoy effect, calculated by subtracting the spike(‐) EV mean infection from the corresponding spike(+) EV mean infection, further detailed in methods. The X‐axis represents normalized S‐LV mean infection levels from the spike(‐) EV condition. The two dotted lines running perpendicular to the X‐axis divide it into thirds, representing the unbiased neutralization sections, with one at x = 33 and one at x = 66. Each data point represents a single dilution from a patient. Data points are colour and symbol coded by their patient of origin. Pearson correlation was performed in order to determine the relationship between the two variables. A linear regression line was overlaid in order to represent this correlation. ‘r’ represents the Pearson correlation coefficient, with a corresponding *P*‐value. (c) Clustered COVID‐19 convalescent serum EV decoy effect data. The Y‐axis represents the EV decoy effect, while the X‐axis groups the data points by the neutralization cluster obtained from panel 5(b). Data are represented as box and whisker plots. The number of data points in each cluster is represented by an ‘n’ value under the box and whiskers plot. Significance was measured by comparing means with one‐way ANOVA followed by Holm‐Sidak multiple comparisons test. ns, not significant; **, *P* ≤ 0.01; ***, *P* ≤ 0.001

We assayed the ability of patient sera to neutralize spike‐mediated viral entry in the presence or absence of spike(+) and spike(‐) EVs. Each serum sample was tested across four different dilutions to cover a broader range of neutralization levels and increase the likelihood of seeing EV‐mediated effects. Neutralization was calculated by normalizing infection levels to infection in the absence of serum. After determining that serum‐mediated neutralization levels correlated positively with anti‐spike and anti‐RBD antibody levels in serum (Supplemental Figure 3), the effect of spike(+) EVs compared to spike(‐) EVs on this neutralization was determined. All nine serum samples demonstrated increasing levels of S‐LV infection as serum was diluted, yielding typical dose‐response curves (Figure 5a – spike(‐) EVs and Supplemental Figure 4). We classified each patient serum sample as low, moderate, or high‐neutralizing based on inhibition of S‐LV entry in the absence of spike(+) EVs, with roughly three patients in each category. Addition of spike(+) EVs led to statistically significant increases in infection—decreased neutralization—for 12 dilutions in five of nine patients. One patient from every neutralization category is shown in Figure 5a with all patient data in Supplemental Figure 4. Without corrections for multiple comparisons, spike(+) EVs increased infections levels at 19 dilutions and in eight patients. Conversely, there were no dilutions for any patients in which infection was significantly higher in the presence of spike(‐) EVs, even without correcting for multiple comparisons.

The reduction of neutralization activity in the presence of spike(+) EVs was most noticeable in the moderate neutralizers, where eight out of 12 tested serum dilutions across patients CPP004, CPP061, and CPP070 had statistically significant increases compared to spike(‐) EVs (Supplemental Figure 4). Although having more variability, low neutralizers too had large increases in infection and reduced neutralization in the presence of spike(+) EVs, suggesting that the spike(+) EV decoy effect is stronger with weak or limiting nAbs. High neutralizers had increased infection in the presence of spike(+) EVs only at higher serum dilutions, suggesting that sufficiently strong or high levels of nAb present at lower serum dilutions can overwhelm the decoy effect of spike(+) EVs.

Next, we plotted the aggregate data from all of the patients and dilutions by converting the data to points on an x,y chart. The X‐axis measures the neutralization activity of the patient's serum at that dilution and the Y‐axis measures the ‘decoy effect’: the decrease in neutralization activity of serum in the presence of spike(+) EVs compared to spike(‐) EVs. Decoy effect values greater than zero indicate that spike(+) EVs led to increased infection and reduced neutralization, while negative values would indicate the opposite. Values close to zero would indicate that spike(+) EVs had no differential effect compared to spike(‐) EVs. When plotted in this aggregate fashion, there is clear evidence of a strong trend towards increased infection and reduced neutralization activity of serum in the presence of spike(+) EVs (Figure 5b). Only four of 36 tested serum conditions had negative values, three of which belonged to CPP006, a high neutralizer with very low infection levels. Furthermore, we observed a significant positive correlation between decreasing neutralization capacity and the magnitude of the observed spike(+) EV decoy effect (Pearson correlation; r = 0.6294, *P* < 0.0001). In other words, the less potent a patient's serum is at neutralizing SARS‐CoV‐2, the greater the ‘decoy effect’ of spike‐expressing EVs, reducing the neutralizing activity further. In contrast, patients with strong neutralizing activity were more resistant to this ‘decoy effect.’ To make intergroup comparisons of the EV decoy effect, we divided the neutralization range into three equal sections, and examined all of the data points—irrespective of initial patient classification—that clustered into strong, moderate, and low neutralizing sections (Figure 5c). The mean EV decoy effect of the low neutralizing cluster was significantly higher than that of the moderate (*P* = 0.0060) and strong (*P* < 0.0001) clusters, but we did not observe differences between moderate and strong clusters (*P* = 0.2981) (Figure 5c). Taken together, these data suggest that spike(+) EVs can serve as decoys for a varied population of patient‐derived nAbs produced as a response to COVID‐19 infection, potentially leading to increased infection and lower neutralization during weak and moderate immune responses.

## 4 DISCUSSION

The recent emergence of SARS‐CoV‐2 in the human population and spread across the globe represents the largest viral pandemic in over a century. SARS‐CoV‐2 is particularly dangerous for people with pre‐existing health conditions including diabetes, obesity, pulmonary disease, and cardiovascular disease. In addition, mortality rate increases drastically with age over 70 and many long‐term care facilities for the elderly have seen extremely high death rates (Richardson et al., 2020). As of December 7th, 2020, SARS‐CoV‐2 had infected at least 67 million people worldwide and has been responsible for over 1.5 million deaths. In this study, we demonstrate that EVs released from cells expressing SARS‐CoV‐2 spike protein can incorporate full‐length spike and serve as decoy targets for neutralizing antibodies (nAbs) in convalescent patient sera, reducing the efficiency of nAbs at preventing viral infection. Several important caveats to our findings should be noted. First, the pseudoviruses employed in this study have lower levels of spike than SARS‐CoV‐2 viruses and defective viral particles that can also scavenge nAbs. Second, the in vitro assessment of neutralization using patient sera fails to recapitulate the complex tissue environment where SARS‐CoV‐2 replicates. The importance of the EV decoy effect in vivo will likely depend on the levels of spike‐containing EVs produced by infected cells, particularly in the lung, and the concentrations of nAbs that reach these anatomical regions where viral replication occurs. Whether the EV decoy effect observed here contributes meaningfully to disease outcomes in patients will require further studies with patient samples.

While innate and cell‐mediated immunity are important in viral infections, the humoral immune system plays a particularly critical role, especially neutralizing antibodies (nAbs) that bind to viral spike or envelope proteins and block infection of cells. Neutralizing antibodies are effective against SARS‐CoV‐2. Early studies isolating nAbs from the sera of recovered patients demonstrated that antibodies targeting the receptor binding domain (RBD) on spike that interacts with ACE2 are effective in protecting rodent and primate animal models from SARS‐CoV‐2 challenge (Kreye et al., 2020; Rogers et al., 2020; Zost et al., 2020). More recently, the success of the Pfizer/BioNTech, Moderna, and AstraZeneca vaccines provide clinical evidence of the efficacy of nAbs in blocking SARS‐CoV‐2 infection in humans. Compared to the straightforward, protective role of nAbs in passive immunotherapies or vaccines, the efficacy of nAbs during ‘natural’ SARS‐CoV‐2 infection is less clear. Neutralizing Abs are present at low levels at days 7–10 following infection, increase to higher titers by 2–3 weeks, and are detectable in most patients following infection (Wang et al., 2020). A direct relationship between nAb titers and disease severity has been reported, with the highest nAb titers found in patients with the most severe disease (Boonyaratanakornkit et al., 2020; Kalkan Yazıcı et al., 2020; Liu et al., 2020). These finding that suggest that the nAb response to SARS‐CoV‐2 during infection may be suboptimal, a hypothesis supported by the clinical successes of convalescent plasma targeting the spike protein (Perotti et al., 2020). Similarly, monoclonal antibody therapy against spike has been shown to decrease viral loads and disease severity in infected animals and recently received emergency use authorization from the FDA (Baum et al., 2020). Together, these data indicate that while nAbs can be extremely effective in protecting against SARS‐CoV‐2, the antibody responses that develop during natural infection are not always capable of containing viral replication and disease progression.

The reduced efficiency of nAbs during infection could reflect viral defenses against antibodies. Due to the importance of neutralizing antibodies in controlling viral infections, viruses have evolved elaborate and sophisticated strategies to disrupt humoral immune responses, including extensive glycosylation of spike and envelope proteins to shield them from nAbs, escape mutations that alter the surface of spike to prevent or reduce nAb binding, and—for some viruses such as HCV and HIV—direct cell‐to‐cell spread that limits viral exposure to nAbs (Bailey et al., 2004; Brimacombe et al., 2011; Law et al., 2016; Lazarevic et al., 2019). Additionally, previous studies have demonstrated that virally‐infected cells can release particles that act as decoys for the humoral immune system. Hepatitis B virus (HBV) releases ‘sub‐viral particles’—small non‐infectious particles that carry the HBV viral fusion glycoprotein, HBsAg—that outnumber infectious viral particles approximately 2000:1 (Gerlich, 2013; Rydell et al., 2017). Other viruses, like HIV, have evolved a built‐in inefficiency – multiple non‐infectious, immature viral particles are released from the infected cell alongside infectious virions (Joyner et al., 2011). These defective viral particles vastly outnumber infectious HIV and are thought to contribute to the dominance of non‐neutralizing Abs during HIV infection by displaying non‐functional conformations of the HIV fusion glycoprotein gp120.

Here, we demonstrate that another class of viral‐host intermediate particles—extracellular vesicles (EVs) bearing viral spike or envelope glycoproteins—can also interact with the immune system and may shape the outcome of viral infections. Nearly all cells produce extracellular vesicles (EVs), and previous studies have demonstrated that EVs can package viral proteins and RNAs (Abels & Breakefield, 2016). Given that EVs and viruses can originate in the endosomal compartments or at interior surface of the plasma membrane and utilize endosomal sorting complexes required for transport (ESCRT) machinery to leave the cell, it is not surprising that EVs containing significant amounts of viral proteins can be detected. It is now becoming apparent that virally‐infected cells release a gradient of different particles spanning the gamut from EVs containing almost entirely host proteins and RNAs to viral particles comprised predominantly of viral material, although even functional, replication‐competent viruses often incorporate many host proteins (Burnie & Guzzo, 2019; Nolte‐‘T Hoen et al., 2016; Shaw et al., 2008). Our finding that EVs containing SARS‐CoV‐2 spike interact with the humoral immune system and reduce serum nAb efficacy reveals a novel property of EVs that adds an important dimension to the growing literature on EVs during viral infections. Interestingly, EVs can have dichotomous roles in this regard. EVs can be used therapeutically to fight viral infection, demonstrated clearly by a recent study showing that engineered EVs containing ACE2 and TMPRSS2 sequestered SARS‐CoV‐2 spike pseudovirus and prevented infection (Cocozza et al., 2020). On the other hand, previous studies have demonstrated that EVs can promote viral infection via several mechanisms, including sensitizing nearby cells for infection, causing bystander immune cells to undergo apoptosis, or completely encapsulating non‐enveloped viruses to shield them from the immune system (Arenaccio et al., 2014; Feng et al., 2013; Gu et al., 2020; Lenassi et al., 2010; Nagashima et al., 2014). Similar EV‐mediated proviral effects were recently postulated to be able to occur in the context of COVID‐19, an idea which our findings support (Askenase, 2020). EVs carrying viral envelope glycoproteins to serve as immune decoys is likely one more EV‐based tool in the viral arsenal for evading host defenses. These spike‐containing EVs are likely acting as a competitive inhibitor of nAbs based upon the dose‐dependent effect and ability of high levels of nAbs to overcome the EVs and fully inhibit viral entry.

Finally, this study highlights the complex and overlapping interactions of EVs, viruses, and the immune system that occur during viral infections. Although our NFC data indicated that the majority of EVs released from spike‐expressing cells do not actually contain spike, we observed a significant inhibitory effect of spike‐containing EVs on commercial and COVID‐19 patient‐derived nAb efficiency. This is more remarkable considering that most of the detectable spike on EVs had shed the S1 subunit, which contains the ACE2 RBD and is target of most nAbs. This suggests that the few EVs containing full‐length spike have an outsized effect in reducing nAb efficacy; or alternatively, nAbs against S2 epitopes are also an important factor in the EV decoy effect. This could explain why we saw more significant spike‐EV decoy effects on patient serum, where there are likely multiple types of anti‐spike antibodies present, compared to the commercial nAb that only targets one epitope on full‐length spike. This ability of EVs to serve as immune decoys, particularly against patient nAbs generated as a response to natural COVID‐19 infection, establishes a novel and previously unreported role of EVs in the context of viral infection. If EVs bearing SARS‐CoV‐2 spike reduce nAb efficacy in vivo as they do in vitro, the interplay between EVs and the immune response could help determine disease outcome. As patients with high neutralizing activity and high concentrations of commercial nAbs were able to fully overcome the inhibitory effect of spike(+) EVs in vitro, we anticipate that if the decoy effect of EVs plays an important role in vivo*—*which, as mentioned above will require further studies with patient samples—it could be overcome by administering additional nAb in the form of monoclonal antibody or convalescent plasma therapy. We do not anticipate that EVs will interfere with nAb efficacy during vaccination, as the cells expressing spike—and any EVs incorporating spike—would likely be cleared prior to exposure to the virus. However, the knowledge of this property of EVs could help inform novel antiviral therapies, such as drugs that target EV release or prevent EV incorporation of viral proteins to increase the effectiveness of nAb responses in passive immunotherapies or during infection. Finally, while this paper has focused on nAbs, non‐neutralizing antibodies also play important roles during viral infection, including mediating complement‐dependent lysis and antibody‐dependent cellular cytotoxicity (ADCC). Further studies will continue to illuminate the complex interactions between EVs and the humoral immune system and how viruses subvert EV pathways to promote their replication, revealing an intricate molecular cat‐and‐mouse game that unfolds among sub‐micron particles and antibodies during viral infection.

## CONFLICTS OF INTEREST

The authors report no conflict of interest.

## Supporting information

Supporting Information

Supporting Information

Supporting Information

Supporting Information

Supporting Information

Supporting Information
