# Supplementary material for: Extracellular vesicles carry SARS‐CoV‐2 spike protein and serve as decoys for neutralizing antibodies
Source: J Extracell Vesicles. 2021 Jun 18;10(8):e12112. doi: 10.1002/jev2.12112 (PMC8213968; doi:10.1002/jev2.12112)
Supplement: Supplementary file 5 — Supporting Information [file JEV2-10-e12112-s006.pdf]

Supplemental File

Mass Spectrometry of spike(+) EVs

2.5 mLs of TFF-purified spike(+) EVs were ultracentrifuged at 110,000g for 2 hours using a SW55Ti rotor to pellet EVs. The EV pellet was lysed with 2% SDS mixed with protease inhibitor tablets (Roche Diagnostics). Detergent was removed by the previously published FASP protocol using a 10-kDa cutoff filter (Millipore), and the buffer exchanged for 8 M urea in 50 mM Tris (pH 8) to a final volume of 50 μL[1]. Fifty microliters of 50mM Tris pH 8.5 was added to EV samples prior to digestion to yield a concentration of 2M Urea. The samples were reduced with 10mM dithiothreitol for 1-hour at 37°C, followed by alkylation with 25mM iodoacetaminde for 30min in the dark. The digestion was performed with 0.3 micrograms of lysyl endopeptidase on for 1 hour at 37°C followed by the addition of 0.3 micrograms of trypsin and incubation overnight on at 37°C. Sample were analyzed by LC-MS/MS using a Orbitrap Eclipse Tribrid mass spectrometer (Thermo Scientific) equipped with a nanoACQUITYTM Ultra-high pressure liquid chromatography system (Waters). Mobile phases were 0.1% formic acid in water (A) and 0.1% formic acid in acetonitrile (B). Peptides were loaded onto a nanoACQUITY UPLC 2G-V/M C18 desalting trap column (180 μm x 20 mm nano column, 5 μm, 100 A˚) at flow rate of 0.300μl/minute. Subsequently, peptides were resolved in a nanoACQUITY UPLC BEH300 C18 reversed phase column (75μm x 250 mm nano column, 1.7μm, 100 A˚; Waters) followed by a gradient elution of 1-50 % of Mobile phase B in 120 minutes. A nano ES ion source at a flow rate of 300 nL/min, 1.5 kV spray voltage, and 270°C capillary temperature was

utilized to ionize peptides. Full scan MS spectra (m/z 380-1800) were acquired at a resolution of 60,000 followed by twenty data dependent MS/MS scans. MS/MS spectra were generated by collision-induced dissociation of the peptide ions (normalized collision energy = 35%; activation Q = 0.250; activation time = 20 ms) to generate a series of b- and y-ions as major fragments. The dynamic exclusion list was confined to a maximum of 500 entries with exclusion duration of 60 seconds and mass accuracy of 10 ppm for the precursor monoisotopic mass. LC-MS/MS raw data were acquired using the Xcalibur software (Thermo Fisher Scientific, version 2.2 SP1). The LC-MS/MS raw files were processed and search using Mascot (Matrix Science London, version 2.1) and searched against the Uniprot database that contained both viral and human entries (75,005 sequences; 25,774,322 residues). Mascot search settings were as follow: trypsin enzyme specificity; mass accuracy window for precursor ion, 8 ppm; mass accuracy window for fragment ions, 0.8 Da; carbamidomethylation of cysteines as fixed modifications; oxidation of methionine as variable modification; and one missed cleavage. Searched files were then imported into Scaffold (Proteome Software Inc., version 4.6.2) for peptide validation and quantification by spectral count. Peptide identifications were accepted if there were >95% probability by the Peptide Prophet algorithm. Spectral counts were reported using total spectrum count for each peptide identified.

References

1. Wiśniewski, J. R., Zougman, A., Nagaraj, N. & Mann, M. Universal sample preparation method for proteome analysis. *Nature Methods* **6**, 359–362 (2009).

#	Identified Proteins (500/586)	Accession Number	Spectral Count
1	Cluster of sp|P78527|PRKDC_HUMAN DNA-dependent protein kinase catalytic subunit OS=Homo sapiens OX=9606 GN=PRKDC PE=1 SV=3 (P78527)	P78527 [2]	35
2	Cluster of sp|P07900|HS90A_HUMAN Heat shock protein HSP 90-alpha OS=Homo sapiens OX=9606 GN=HSP90AA1 PE=1 SV=5 (P07900)	P07900 [7]	30
3	Cluster of sp|Q07954|LRP1_HUMAN Prolow-density lipoprotein receptor-related protein 1 OS=Homo sapiens OX=9606 GN=LRP1 PE=1 SV=2 (Q07954)	Q07954 [2]	27
4	Cluster of sp|Q9Y490|TLN1_HUMAN Talin-1 OS=Homo sapiens OX=9606 GN=TLN1 PE=1 SV=3 (Q9Y490)	Q9Y490 [3]	25
5	Cluster of sp|Q9BUF5|TBB6_HUMAN Tubulin beta-6 chain OS=Homo sapiens OX=9606 GN=TUBB6 PE=1 SV=1 (Q9BUF5)	Q9BUF5 [11]	25
6	Cluster of sp|Q14204|DYHC1_HUMAN Cytoplasmic dynein 1 heavy chain 1 OS=Homo sapiens OX=9606 GN=DYNC1H1 PE=1 SV=5 (Q14204)	Q14204 [2]	23
7	Cluster of tr|A0A087WVQ6|A0A087WVQ6_HUMAN Clathrin heavy chain OS=Homo sapiens OX=9606 GN=CLTC PE=1 SV=1 (A0A087WVQ6)	A0A087WVQ6 [3]	21
8	Cluster of sp|P09874|PARP1_HUMAN Poly [ADP-ribose] polymerase 1 OS=Homo sapiens OX=9606 GN=PARP1 PE=1 SV=4 (P09874)	P09874 [2]	18
9	Cluster of tr|A0A0G2JIW1|A0A0G2JIW1_HUMAN Heat shock 70 kDa protein 1B OS=Homo sapiens OX=9606 GN=HSPA1B PE=1 SV=1 (A0A0G2JIW1)	A0A0G2JIW1 [4]	16
10	sp|P07996|TSP1_HUMAN Thrombospondin-1 OS=Homo sapiens OX=9606 GN=THBS1 PE=1 SV=2	P07996	15
11	sp|P13639|EF2_HUMAN Elongation factor 2 OS=Homo sapiens OX=9606 GN=EEF2 PE=1 SV=4	P13639	15
12	sp|P04264|K2C1_HUMAN Keratin, type II cytoskeletal 1 OS=Homo sapiens OX=9606 GN=KRT1 PE=1 SV=6	P04264	15
13	Cluster of sp|P13645|K1C10_HUMAN Keratin, type I cytoskeletal 10 OS=Homo sapiens OX=9606 GN=KRT10 PE=1 SV=6 (P13645)	P13645 [11]	14
14	sp|P02751|FINC_HUMAN Fibronectin OS=Homo sapiens OX=9606 GN=FN1 PE=1 SV=5	P02751	14
15	Cluster of sp|P68366|TBA4A_HUMAN Tubulin alpha-4A chain OS=Homo sapiens OX=9606 GN=TUBA4A PE=1 SV=1 (P68366)	P68366 [7]	14
16	Cluster of sp|P50990|TCPQ_HUMAN T-complex protein 1 subunit theta OS=Homo sapiens OX=9606 GN=CCT8 PE=1 SV=4 (P50990)	P50990 [3]	14
17	sp|P00734|THRB_HUMAN Prothrombin OS=Homo sapiens OX=9606 GN=F2 PE=1 SV=2	P00734	13
18	Cluster of sp|P11142|HSP7C_HUMAN Heat shock cognate 71 kDa protein OS=Homo sapiens OX=9606 GN=HSPA8 PE=1 SV=1 (P11142)	P11142 [4]	13
19	tr|A0A0U1RQF0|A0A0U1RQF0_HUMAN Fatty acid synthase OS=Homo sapiens OX=9606 GN=FASN PE=1 SV=1	A0A0U1RQF0 (+1)	12
20	sp|P07814|SYEP_HUMAN Bifunctional glutamate/proline--tRNA ligase OS=Homo sapiens OX=9606 GN=EPRS1 PE=1 SV=5	P07814	12
21	sp|Q99832|TCPH_HUMAN T-complex protein 1 subunit eta OS=Homo sapiens OX=9606 GN=CCT7 PE=1 SV=2	Q99832	12
22	sp|P55072|TERA_HUMAN Transitional endoplasmic reticulum ATPase OS=Homo sapiens OX=9606 GN=VCP PE=1 SV=4	P55072	11
23	Cluster of sp|P31946|1433B_HUMAN 14-3-3 protein beta/alpha OS=Homo sapiens OX=9606 GN=YWHAB PE=1 SV=3 (P31946)	P31946 [4]	11
24	sp|P11021|BIP_HUMAN Endoplasmic reticulum chaperone BiP OS=Homo sapiens OX=9606 GN=HSPA5 PE=1 SV=2	P11021	11
25	Cluster of sp|P21333|FLNA_HUMAN Filamin-A OS=Homo sapiens OX=9606 GN=FLNA PE=1 SV=4 (P21333)	P21333 [6]	10
26	Cluster of sp|P14618|KPYM_HUMAN Pyruvate kinase PKM OS=Homo sapiens OX=9606 GN=PKM PE=1 SV=4 (P14618)	P14618 [3]	10
27	sp|P19823|ITIH2_HUMAN Inter-alpha-trypsin inhibitor heavy chain H2 OS=Homo sapiens OX=9606 GN=ITIH2 PE=1 SV=2	P19823 (+1)	10
28	sp|P48643|TCPE_HUMAN T-complex protein 1 subunit epsilon OS=Homo sapiens OX=9606 GN=CCT5 PE=1 SV=1	P48643	10
29	sp|Q08211|DHX9_HUMAN ATP-dependent RNA helicase A OS=Homo sapiens OX=9606 GN=DHX9 PE=1 SV=4	Q08211	10
30	sp|P23526|SAHH_HUMAN Adenosylhomocysteinase OS=Homo sapiens OX=9606 GN=AHCY PE=1 SV=4	P23526	10
31	Cluster of sp|P68104|EF1A1_HUMAN Elongation factor 1-alpha 1 OS=Homo sapiens OX=9606 GN=EEF1A1 PE=1 SV=1 (P68104)	P68104 [3]	10
32	sp|P20742|PZP_HUMAN Pregnancy zone protein OS=Homo sapiens OX=9606 GN=PZP PE=1 SV=4	P20742	10
33	sp|P01023|A2MG_HUMAN Alpha-2-macroglobulin OS=Homo sapiens OX=9606 GN=A2M PE=1 SV=3	P01023	9
34	Cluster of sp|P61106|RAB14_HUMAN Ras-related protein Rab-14 OS=Homo sapiens OX=9606 GN=RAB14 PE=1 SV=4 (P61106)	P61106 [15]	9
35	sp|P01024|CO3_HUMAN Complement C3 OS=Homo sapiens OX=9606 GN=C3 PE=1 SV=2	P01024	9
36	tr|A0A0A0MS51|A0A0A0MS51_HUMAN Gelsolin OS=Homo sapiens OX=9606 GN=GSN PE=1 SV=1	A0A0A0MS51 (+2)	9
37	Cluster of sp|P35527|K1C9_HUMAN Keratin, type I cytoskeletal 9 OS=Homo sapiens OX=9606 GN=KRT9 PE=1 SV=3 (P35527)	P35527 [2]	9
38	Cluster of tr|G3XAP6|G3XAP6_HUMAN Cartilage oligomeric matrix protein OS=Homo sapiens OX=9606 GN=COMP PE=1 SV=1 (G3XAP6)	G3XAP6 [4]	9
39	Cluster of sp|P17987|TCPA_HUMAN T-complex protein 1 subunit alpha OS=Homo sapiens OX=9606 GN=TCP1 PE=1 SV=1 (P17987)	P17987 [4]	9
40	Cluster of sp|P35908|K22E_HUMAN Keratin, type II cytoskeletal 2 epidermal OS=Homo sapiens OX=9606 GN=KRT2 PE=1 SV=2 (P35908)	P35908 [3]	9
41	sp|P62593|BLAT_ECOLX Beta-lactamase TEM OS=Escherichia coli OX=562 GN=bla PE=1 SV=1	P62593	9
42	sp|P0DTC2|SPIKE_SARS2 Spike glycoprotein OS=Severe acute respiratory syndrome coronavirus 2 OX=2697049 GN=S PE=1 SV=1	P0DTC2	9
43	sp|P10809|CH60_HUMAN 60 kDa heat shock protein, mitochondrial OS=Homo sapiens OX=9606 GN=HSPD1 PE=1 SV=2	P10809	9
44	Cluster of sp|P35579|MYH9_HUMAN Myosin-9 OS=Homo sapiens OX=9606 GN=MYH9 PE=1 SV=4 (P35579)	P35579 [5]	8
45	tr|A0A0A0MRJ7|A0A0A0MRJ7_HUMAN Coagulation factor V OS=Homo sapiens OX=9606 GN=F5 PE=1 SV=1	A0A0A0MRJ7 (+1)	8
46	Cluster of sp|P49368|TCPG_HUMAN T-complex protein 1 subunit gamma OS=Homo sapiens OX=9606 GN=CCT3 PE=1 SV=4 (P49368)	P49368 [3]	8
47	Cluster of tr|J3KSZ0|J3KSZ0_HUMAN Eukaryotic initiation factor 4A-I (Fragment) OS=Homo sapiens OX=9606 GN=EIF4A1 PE=1 SV=1 (J3KSZ0)	J3KSZ0 [4]	8
48	Cluster of sp|P14868|SYDC_HUMAN Aspartate--tRNA ligase, cytoplasmic OS=Homo sapiens OX=9606 GN=DARS1 PE=1 SV=2 (P14868)	P14868 [2]	8
49	Cluster of sp|Q14974|IMB1_HUMAN Importin subunit beta-1 OS=Homo sapiens OX=9606 GN=KPNB1 PE=1 SV=2 (Q14974)	Q14974 [3]	8
50	sp|P61247|RS3A_HUMAN 40S ribosomal protein S3a OS=Homo sapiens OX=9606 GN=RPS3A PE=1 SV=2	P61247	8
51	sp|Q9Y230|RUVB2_HUMAN RuvB-like 2 OS=Homo sapiens OX=9606 GN=RUVBL2 PE=1 SV=3	Q9Y230	8
52	sp|Q06033|ITIH3_HUMAN Inter-alpha-trypsin inhibitor heavy chain H3 OS=Homo sapiens OX=9606 GN=ITIH3 PE=1 SV=2	Q06033	7
53	Cluster of sp|P05023|AT1A1_HUMAN Sodium/potassium-transporting ATPase subunit alpha-1 OS=Homo sapiens OX=9606 GN=ATP1A1 PE=1 SV=1 (P05023)	P05023 [7]	7
54	Cluster of sp|P53621|COPA_HUMAN Coatomer subunit alpha OS=Homo sapiens OX=9606 GN=COPA PE=1 SV=2 (P53621)	P53621 [2]	7
55	sp|O75533|SF3B1_HUMAN Splicing factor 3B subunit 1 OS=Homo sapiens OX=9606 GN=SF3B1 PE=1 SV=3	O75533	7
56	sp|Q02878|RL6_HUMAN 60S ribosomal protein L6 OS=Homo sapiens OX=9606 GN=RPL6 PE=1 SV=3	Q02878	7
57	sp|P62241|RS8_HUMAN 40S ribosomal protein S8 OS=Homo sapiens OX=9606 GN=RPS8 PE=1 SV=2	P62241 (+1)	7
58	sp|P14625|ENPL_HUMAN Endoplasmin OS=Homo sapiens OX=9606 GN=HSP90B1 PE=1 SV=1	P14625	7
59	sp|P54289|CA2D1_HUMAN Voltage-dependent calcium channel subunit alpha-2/delta-1 OS=Homo sapiens OX=9606 GN=CACNA2D1 PE=1 SV=3	P54289	7
60	sp|P62805|H4_HUMAN Histone H4 OS=Homo sapiens OX=9606 GN=H4C1 PE=1 SV=2	P62805	7
61	Cluster of sp|P27824|CALX_HUMAN Calnexin OS=Homo sapiens OX=9606 GN=CANX PE=1 SV=2 (P27824)	P27824 [2]	7
62	Cluster of sp|P62258|1433E_HUMAN 14-3-3 protein epsilon OS=Homo sapiens OX=9606 GN=YWHAE PE=1 SV=1 (P62258)	P62258 [2]	7
63	Cluster of tr|F8VPD4|F8VPD4_HUMAN CAD protein OS=Homo sapiens OX=9606 GN=CAD PE=1 SV=1 (F8VPD4)	F8VPD4 [3]	6
64	sp|P04406|G3P_HUMAN Glyceraldehyde-3-phosphate dehydrogenase OS=Homo sapiens OX=9606 GN=GAPDH PE=1 SV=3	P04406	6
65	Cluster of sp|Q99460|PSMD1_HUMAN 26S proteasome non-ATPase regulatory subunit 1 OS=Homo sapiens OX=9606 GN=PSMD1 PE=1 SV=2 (Q99460)	Q99460 [3]	6
66	sp|Q9Y5B9|SP16H_HUMAN FACT complex subunit SPT16 OS=Homo sapiens OX=9606 GN=SUPT16H PE=1 SV=1	Q9Y5B9	6
67	Cluster of sp|P39023|RL3_HUMAN 60S ribosomal protein L3 OS=Homo sapiens OX=9606 GN=RPL3 PE=1 SV=2 (P39023)	P39023 [3]	6
68	Cluster of sp|P30153|2AAA_HUMAN Serine/threonine-protein phosphatase 2A 65 kDa regulatory subunit A alpha isoform OS=Homo sapiens OX=9606 GN=PPP2R1A PE=1 SV=4 (P30153)	P30153 [2]	6
69	sp|P40227|TCPZ_HUMAN T-complex protein 1 subunit zeta OS=Homo sapiens OX=9606 GN=CCT6A PE=1 SV=3	P40227	6

70	sp|P53396|ACLY_HUMAN ATP-citrate synthase OS=Homo sapiens OX=9606 GN=ACLY PE=1 SV=3	P53396	6
71	Cluster of tr|A0A0C4DGB6|A0A0C4DGB6_HUMAN Serum albumin OS=Homo sapiens OX=9606 GN=ALB PE=1 SV=1 (A0A0C4DGB6)	A0A0C4DGB6 [4]	6
72	Cluster of sp|P60228|EIF3E_HUMAN Eukaryotic translation initiation factor 3 subunit E OS=Homo sapiens OX=9606 GN=EIF3E PE=1 SV=1 (P60228)	P60228 [2]	6
73	Cluster of sp|Q76LX8|ATS13_HUMAN A disintegrin and metalloproteinase with thrombospondin motifs 13 OS=Homo sapiens OX=9606 GN=ADAMTS13 PE=1 SV=1 (Q76LX8)	Q76LX8 [2]	6
74	Cluster of tr|A0A2C9F2M7|A0A2C9F2M7_HUMAN D-3-phosphoglycerate dehydrogenase OS=Homo sapiens OX=9606 GN=PHGDH PE=1 SV=1 (A0A2C9F2M7)	A0A2C9F2M7 [5]	6
75	sp|P19827|ITIH1_HUMAN Inter-alpha-trypsin inhibitor heavy chain H1 OS=Homo sapiens OX=9606 GN=ITIH1 PE=1 SV=3	P19827	6
76	sp|P22314|UBA1_HUMAN Ubiquitin-like modifier-activating enzyme 1 OS=Homo sapiens OX=9606 GN=UBA1 PE=1 SV=3	P22314	6
77	tr|A8MUD9|A8MUD9_HUMAN 60S ribosomal protein L7 OS=Homo sapiens OX=9606 GN=RPL7 PE=1 SV=1	A8MUD9 (+1)	6
78	Cluster of tr|R4GNH3|R4GNH3_HUMAN 26S proteasome regulatory subunit 6A OS=Homo sapiens OX=9606 GN=PSMC3 PE=1 SV=1 (R4GNH3)	R4GNH3 [2]	6
79	Cluster of tr|E9PCY7|E9PCY7_HUMAN Heterogeneous nuclear ribonucleoprotein H OS=Homo sapiens OX=9606 GN=HNRNPH1 PE=1 SV=1 (E9PCY7)	E9PCY7 [5]	6
80	Cluster of sp|P19338|NUCL_HUMAN Nucleolin OS=Homo sapiens OX=9606 GN=NCL PE=1 SV=3 (P19338)	P19338 [2]	6
81	Cluster of sp|Q9Y265|RUVB1_HUMAN RuvB-like 1 OS=Homo sapiens OX=9606 GN=RUVBL1 PE=1 SV=1 (Q9Y265)	Q9Y265 [2]	6
82	sp|P26373|RL13_HUMAN 60S ribosomal protein L13 OS=Homo sapiens OX=9606 GN=RPL13 PE=1 SV=4	P26373	6
83	Cluster of sp|P69905|HBA_HUMAN Hemoglobin subunit alpha OS=Homo sapiens OX=9606 GN=HBA1 PE=1 SV=2 (P69905)	P69905 [2]	5
84	Cluster of tr|A0A087X0X3|A0A087X0X3_HUMAN Heterogeneous nuclear ribonucleoprotein M OS=Homo sapiens OX=9606 GN=HNRNPM PE=1 SV=1 (A0A087X0X3)	A0A087X0X3 [4]	5
85	sp|P78371|TCPB_HUMAN T-complex protein 1 subunit beta OS=Homo sapiens OX=9606 GN=CCT2 PE=1 SV=4	P78371	5
86	tr|I3L1L3|I3L1L3_HUMAN Myb-binding protein 1A (Fragment) OS=Homo sapiens OX=9606 GN=MYBBP1A PE=1 SV=1	I3L1L3 (+1)	5
87	tr|A0A0A0MSX9|A0A0A0MSX9_HUMAN Isoleucine--tRNA ligase, cytoplasmic OS=Homo sapiens OX=9606 GN=IARS1 PE=1 SV=1	A0A0A0MSX9 (+1)	5
88	sp|Q12860|CNTN1_HUMAN Contactin-1 OS=Homo sapiens OX=9606 GN=CNTN1 PE=1 SV=1	Q12860	5
89	sp|P36578|RL4_HUMAN 60S ribosomal protein L4 OS=Homo sapiens OX=9606 GN=RPL4 PE=1 SV=5	P36578	5
90	Cluster of sp|P00558|PGK1_HUMAN Phosphoglycerate kinase 1 OS=Homo sapiens OX=9606 GN=PGK1 PE=1 SV=3 (P00558)	P00558 [2]	5
91	Cluster of tr|A0A5F9ZHM4|A0A5F9ZHM4_HUMAN L-lactate dehydrogenase OS=Homo sapiens OX=9606 GN=LDHB PE=1 SV=1 (A0A5F9ZHM4)	A0A5F9ZHM4 [9]	5
92	Cluster of sp|Q00839|HNRPU_HUMAN Heterogeneous nuclear ribonucleoprotein U OS=Homo sapiens OX=9606 GN=HNRNPU PE=1 SV=6 (Q00839)	Q00839 [2]	5
93	Cluster of sp|P62701|RS4X_HUMAN 40S ribosomal protein S4, X isoform OS=Homo sapiens OX=9606 GN=RPS4X PE=1 SV=2 (P62701)	P62701 [2]	5
94	sp|P06737|PYGL_HUMAN Glycogen phosphorylase, liver form OS=Homo sapiens OX=9606 GN=PYGL PE=1 SV=4	P06737	5
95	Cluster of sp|P50395|GDIB_HUMAN Rab GDP dissociation inhibitor beta OS=Homo sapiens OX=9606 GN=GDI2 PE=1 SV=2 (P50395)	P50395 [2]	5
96	Cluster of sp|Q15393|SF3B3_HUMAN Splicing factor 3B subunit 3 OS=Homo sapiens OX=9606 GN=SF3B3 PE=1 SV=4 (Q15393)	Q15393 [2]	5
97	sp|P25786|PSA1_HUMAN Proteasome subunit alpha type-1 OS=Homo sapiens OX=9606 GN=PSMA1 PE=1 SV=1	P25786	5
98	sp|P26641|EF1G_HUMAN Elongation factor 1-gamma OS=Homo sapiens OX=9606 GN=EEF1G PE=1 SV=3	P26641	5
99	sp|P62269|RS18_HUMAN 40S ribosomal protein S18 OS=Homo sapiens OX=9606 GN=RPS18 PE=1 SV=3	P62269	5
100	sp|P07737|PROF1_HUMAN Profilin-1 OS=Homo sapiens OX=9606 GN=PFN1 PE=1 SV=2	P07737	5
101	Cluster of sp|P62244|RS15A_HUMAN 40S ribosomal protein S15a OS=Homo sapiens OX=9606 GN=RPS15A PE=1 SV=2 (P62244)	P62244 [2]	5
102	Cluster of sp|P84085|ARF5_HUMAN ADP-ribosylation factor 5 OS=Homo sapiens OX=9606 GN=ARF5 PE=1 SV=2 (P84085)	P84085 [4]	5
103	tr|C9JNW5|C9JNW5_HUMAN 60S ribosomal protein L24 OS=Homo sapiens OX=9606 GN=RPL24 PE=1 SV=1	C9JNW5 (+2)	5
104	Cluster of sp|P12111|CO6A3_HUMAN Collagen alpha-3(VI) chain OS=Homo sapiens OX=9606 GN=COL6A3 PE=1 SV=5 (P12111)	P12111 [2]	5
105	sp|Q9UBG0|MRC2_HUMAN C-type mannose receptor 2 OS=Homo sapiens OX=9606 GN=MRC2 PE=1 SV=2	Q9UBG0	4
106	Cluster of sp|P04114|APOB_HUMAN Apolipoprotein B-100 OS=Homo sapiens OX=9606 GN=APOB PE=1 SV=2 (P04114)	P04114 [2]	4
107	Cluster of sp|Q5T4S7|UBR4_HUMAN E3 ubiquitin-protein ligase UBR4 OS=Homo sapiens OX=9606 GN=UBR4 PE=1 SV=1 (Q5T4S7)	Q5T4S7 [2]	4
108	Cluster of sp|Q6P2Q9|PRP8_HUMAN Pre-mRNA-processing-splicing factor 8 OS=Homo sapiens OX=9606 GN=PRPF8 PE=1 SV=2 (Q6P2Q9)	Q6P2Q9 [2]	4
109	sp|Q13200|PSMD2_HUMAN 26S proteasome non-ATPase regulatory subunit 2 OS=Homo sapiens OX=9606 GN=PSMD2 PE=1 SV=3	Q13200	4
110	sp|O75643|U520_HUMAN U5 small nuclear ribonucleoprotein 200 kDa helicase OS=Homo sapiens OX=9606 GN=SNRNP200 PE=1 SV=2	O75643	4
111	sp|P13010|XRCC5_HUMAN X-ray repair cross-complementing protein 5 OS=Homo sapiens OX=9606 GN=XRCC5 PE=1 SV=3	P13010	4
112	tr|F5GZS6|F5GZS6_HUMAN 4F2 cell-surface antigen heavy chain OS=Homo sapiens OX=9606 GN=SLC3A2 PE=1 SV=1	F5GZS6 (+2)	4
113	sp|P50991|TCPD_HUMAN T-complex protein 1 subunit delta OS=Homo sapiens OX=9606 GN=CCT4 PE=1 SV=4	P50991	4
114	Cluster of tr|A0A0U1RR32|A0A0U1RR32_HUMAN Histone H2A OS=Homo sapiens OX=9606 GN=hCG_2039566 PE=3 SV=1 (A0A0U1RR32)	A0A0U1RR32 [14]	4
115	sp|P12956|XRCC6_HUMAN X-ray repair cross-complementing protein 6 OS=Homo sapiens OX=9606 GN=XRCC6 PE=1 SV=2	P12956	4
116	Cluster of sp|P23142|FBLN1_HUMAN Fibulin-1 OS=Homo sapiens OX=9606 GN=FBLN1 PE=1 SV=4 (P23142)	P23142 [2]	4
117	Cluster of sp|Q9UBF2|COPG2_HUMAN Coatomer subunit gamma-2 OS=Homo sapiens OX=9606 GN=COPG2 PE=1 SV=1 (Q9UBF2)	Q9UBF2 [2]	4
118	tr|X6RJP6|X6RJP6_HUMAN Transgelin-2 (Fragment) OS=Homo sapiens OX=9606 GN=TAGLN2 PE=1 SV=1	X6RJP6	4
119	Cluster of tr|H0YJM8|H0YJM8_HUMAN Proteasome subunit beta type-5 (Fragment) OS=Homo sapiens OX=9606 GN=PSMB5 PE=1 SV=1 (H0YJM8)	H0YJM8 [2]	4
120	Cluster of sp|O14818|PSA7_HUMAN Proteasome subunit alpha type-7 OS=Homo sapiens OX=9606 GN=PSMA7 PE=1 SV=1 (O14818)	O14818 [2]	4
121	sp|O43242|PSMD3_HUMAN 26S proteasome non-ATPase regulatory subunit 3 OS=Homo sapiens OX=9606 GN=PSMD3 PE=1 SV=2	O43242	4
122	sp|P35606|COPB2_HUMAN Coatomer subunit beta' OS=Homo sapiens OX=9606 GN=COPB2 PE=1 SV=2	P35606	4
123	Cluster of tr|A0A494C165|A0A494C165_HUMAN Xaa-Pro dipeptidase (Fragment) OS=Homo sapiens OX=9606 GN=PEPD PE=1 SV=1 (A0A494C165)	A0A494C165 [2]	4
124	tr|E7EPB3|E7EPB3_HUMAN 60S ribosomal protein L14 OS=Homo sapiens OX=9606 GN=RPL14 PE=1 SV=1	E7EPB3	4
125	Cluster of tr|F8VU65|F8VU65_HUMAN 60S acidic ribosomal protein P0 (Fragment) OS=Homo sapiens OX=9606 GN=RPLP0 PE=1 SV=1 (F8VU65)	F8VU65 [5]	4
126	sp|P11047|LAMC1_HUMAN Laminin subunit gamma-1 OS=Homo sapiens OX=9606 GN=LAMC1 PE=1 SV=3	P11047	4
127	sp|P25788|PSA3_HUMAN Proteasome subunit alpha type-3 OS=Homo sapiens OX=9606 GN=PSMA3 PE=1 SV=2	P25788	4
128	Cluster of sp|P25789|PSA4_HUMAN Proteasome subunit alpha type-4 OS=Homo sapiens OX=9606 GN=PSMA4 PE=1 SV=1 (P25789)	P25789 [2]	4
129	sp|P04746|AMYP_HUMAN Pancreatic alpha-amylase OS=Homo sapiens OX=9606 GN=AMY2A PE=1 SV=2	P04746	4
130	sp|Q7L2H7|EIF3M_HUMAN Eukaryotic translation initiation factor 3 subunit M OS=Homo sapiens OX=9606 GN=EIF3M PE=1 SV=1	Q7L2H7	4
131	tr|A0A024RA52|A0A024RA52_HUMAN Proteasome subunit alpha type OS=Homo sapiens OX=9606 GN=PSMA2 PE=1 SV=1	A0A024RA52 (+1)	4
132	tr|A0A087X2I1|A0A087X2I1_HUMAN 26S proteasome regulatory subunit 10B OS=Homo sapiens OX=9606 GN=PSMC6 PE=1 SV=1	A0A087X2I1 (+1)	4
133	tr|A0A499FI48|A0A499FI48_HUMAN Protein disulfide-isomerase OS=Homo sapiens OX=9606 GN=PDIA4 PE=1 SV=1	A0A499FI48	4
134	sp|Q12907|LMAN2_HUMAN Vesicular integral-membrane protein VIP36 OS=Homo sapiens OX=9606 GN=LMAN2 PE=1 SV=1	Q12907	4
135	tr|E5RI99|E5RI99_HUMAN 60S ribosomal protein L30 (Fragment) OS=Homo sapiens OX=9606 GN=RPL30 PE=1 SV=1	E5RI99 (+1)	4
136	sp|P01031|CO5_HUMAN Complement C5 OS=Homo sapiens OX=9606 GN=C5 PE=1 SV=4	P01031	4
137	sp|P35998|PRS7_HUMAN 26S proteasome regulatory subunit 7 OS=Homo sapiens OX=9606 GN=PSMC2 PE=1 SV=3	P35998	4
138	Cluster of sp|P61353|RL27_HUMAN 60S ribosomal protein L27 OS=Homo sapiens OX=9606 GN=RPL27 PE=1 SV=2 (P61353)	P61353 [2]	4
139	sp|Q9Y3U8|RL36_HUMAN 60S ribosomal protein L36 OS=Homo sapiens OX=9606 GN=RPL36 PE=1 SV=3	Q9Y3U8	4
140	sp|P62195|PRS8_HUMAN 26S proteasome regulatory subunit 8 OS=Homo sapiens OX=9606 GN=PSMC5 PE=1 SV=1	P62195	4

141	sp|P06732|KCRM_HUMAN Creatine kinase M-type OS=Homo sapiens OX=9606 GN=CKM PE=1 SV=2	P06732	4
142	sp|P18085|ARF4_HUMAN ADP-ribosylation factor 4 OS=Homo sapiens OX=9606 GN=ARF4 PE=1 SV=3	P18085	4
143	sp|Q9P2J5|SYLC_HUMAN Leucine--tRNA ligase, cytoplasmic OS=Homo sapiens OX=9606 GN=LARS1 PE=1 SV=2	Q9P2J5	4
144	sp|P62424|RL7A_HUMAN 60S ribosomal protein L7a OS=Homo sapiens OX=9606 GN=RPL7A PE=1 SV=2	P62424	4
145	sp|Q13347|EIF3I_HUMAN Eukaryotic translation initiation factor 3 subunit I OS=Homo sapiens OX=9606 GN=EIF3I PE=1 SV=1	Q13347	4
146	sp|P30101|PDIA3_HUMAN Protein disulfide-isomerase A3 OS=Homo sapiens OX=9606 GN=PDIA3 PE=1 SV=4	P30101	4
147	sp|Q8NI27|THOC2_HUMAN THO complex subunit 2 OS=Homo sapiens OX=9606 GN=THOC2 PE=1 SV=2	Q8NI27	4
148	sp|Q14152|EIF3A_HUMAN Eukaryotic translation initiation factor 3 subunit A OS=Homo sapiens OX=9606 GN=EIF3A PE=1 SV=1	Q14152	3
149	sp|B5ME19|EIFCL_HUMAN Eukaryotic translation initiation factor 3 subunit C-like protein OS=Homo sapiens OX=9606 GN=EIF3CL PE=1 SV=1	B5ME19 (+1)	3
150	sp|Q9NR30|DDX21_HUMAN Nucleolar RNA helicase 2 OS=Homo sapiens OX=9606 GN=DDX21 PE=1 SV=5	Q9NR30	3
151	Cluster of tr|A0A0G2JPR0|A0A0G2JPR0_HUMAN Complement C4-A OS=Homo sapiens OX=9606 GN=C4A PE=1 SV=1 (A0A0G2JPR0)	A0A0G2JPR0 [6]	3
152	Cluster of tr|G3XAI2|G3XAI2_HUMAN Laminin subunit beta-1 OS=Homo sapiens OX=9606 GN=LAMB1 PE=1 SV=1 (G3XAI2)	G3XAI2 [3]	3
153	sp|P55884|EIF3B_HUMAN Eukaryotic translation initiation factor 3 subunit B OS=Homo sapiens OX=9606 GN=EIF3B PE=1 SV=3	P55884	3
154	Cluster of sp|Q15008|PSMD6_HUMAN 26S proteasome non-ATPase regulatory subunit 6 OS=Homo sapiens OX=9606 GN=PSMD6 PE=1 SV=1 (Q15008)	Q15008 [2]	3
155	Cluster of tr|A0A0A0MSQ0|A0A0A0MSQ0_HUMAN Plastin-3 OS=Homo sapiens OX=9606 GN=PLS3 PE=1 SV=1 (A0A0A0MSQ0)	A0A0A0MSQ0 [3]	3
156	tr|A0A087X0S5|A0A087X0S5_HUMAN Collagen alpha-1(VI) chain OS=Homo sapiens OX=9606 GN=COL6A1 PE=1 SV=1	A0A087X0S5 (+1)	3
157	tr|A0A0C4DGH5|A0A0C4DGH5_HUMAN Cullin-associated NEDD8-dissociated protein 1 (Fragment) OS=Homo sapiens OX=9606 GN=CAND1 PE=1 SV=1	A0A0C4DGH5 (+1)	3
158	Cluster of tr|G3V2Q1|G3V2Q1_HUMAN Heterogeneous nuclear ribonucleoproteins C1/C2 OS=Homo sapiens OX=9606 GN=HNRNPC PE=1 SV=1 (G3V2Q1)	G3V2Q1 [4]	3
159	Cluster of sp|Q06830|PRDX1_HUMAN Peroxiredoxin-1 OS=Homo sapiens OX=9606 GN=PRDX1 PE=1 SV=1 (Q06830)	Q06830 [2]	3
160	Cluster of sp|Q08945|SSRP1_HUMAN FACT complex subunit SSRP1 OS=Homo sapiens OX=9606 GN=SSRP1 PE=1 SV=1 (Q08945)	Q08945 [2]	3
161	Cluster of tr|K7EPF6|K7EPF6_HUMAN 6-phosphogluconate dehydrogenase, decarboxylating (Fragment) OS=Homo sapiens OX=9606 GN=PGD PE=1 SV=1 (K7EPF6)	K7EPF6 [2]	3
162	sp|P46776|RL27A_HUMAN 60S ribosomal protein L27a OS=Homo sapiens OX=9606 GN=RPL27A PE=1 SV=2	P46776	3
163	Cluster of sp|Q13263|TIF1B_HUMAN Transcription intermediary factor 1-beta OS=Homo sapiens OX=9606 GN=TRIM28 PE=1 SV=5 (Q13263)	Q13263 [2]	3
164	tr|H0YHA7|H0YHA7_HUMAN 60S ribosomal protein L18 (Fragment) OS=Homo sapiens OX=9606 GN=RPL18 PE=1 SV=1	H0YHA7	3
165	sp|P61313|RL15_HUMAN 60S ribosomal protein L15 OS=Homo sapiens OX=9606 GN=RPL15 PE=1 SV=2	P61313	3
166	tr|G3V295|G3V295_HUMAN Proteasome subunit alpha type OS=Homo sapiens OX=9606 GN=PSMA6 PE=1 SV=1	G3V295 (+2)	3
167	sp|O00232|PSD12_HUMAN 26S proteasome non-ATPase regulatory subunit 12 OS=Homo sapiens OX=9606 GN=PSMD12 PE=1 SV=3	O00232	3
168	Cluster of sp|P05387|RLA2_HUMAN 60S acidic ribosomal protein P2 OS=Homo sapiens OX=9606 GN=RPLP2 PE=1 SV=1 (P05387)	P05387 [2]	3
169	sp|P54136|SYRC_HUMAN Arginine--tRNA ligase, cytoplasmic OS=Homo sapiens OX=9606 GN=RARS1 PE=1 SV=2	P54136	3
170	tr|A0A0B4J1R4|A0A0B4J1R4_HUMAN 4-hydroxyphenylpyruvate dioxygenase OS=Homo sapiens OX=9606 GN=HPD PE=1 SV=1	A0A0B4J1R4 (+1)	3
171	Cluster of sp|P36955|PEDF_HUMAN Pigment epithelium-derived factor OS=Homo sapiens OX=9606 GN=SERPINF1 PE=1 SV=4 (P36955)	P36955 [2]	3
172	sp|P49721|PSB2_HUMAN Proteasome subunit beta type-2 OS=Homo sapiens OX=9606 GN=PSMB2 PE=1 SV=1	P49721	3
173	sp|P56192|SYMC_HUMAN Methionine--tRNA ligase, cytoplasmic OS=Homo sapiens OX=9606 GN=MARS1 PE=1 SV=2	P56192	3
174	sp|Q9UHB9|SRP68_HUMAN Signal recognition particle subunit SRP68 OS=Homo sapiens OX=9606 GN=SRP68 PE=1 SV=2	Q9UHB9	3
175	tr|M0R0F0|M0R0F0_HUMAN 40S ribosomal protein S5 (Fragment) OS=Homo sapiens OX=9606 GN=RPS5 PE=1 SV=1	M0R0F0 (+2)	3
176	sp|P28066|PSA5_HUMAN Proteasome subunit alpha type-5 OS=Homo sapiens OX=9606 GN=PSMA5 PE=1 SV=3	P28066	3
177	sp|P62081|RS7_HUMAN 40S ribosomal protein S7 OS=Homo sapiens OX=9606 GN=RPS7 PE=1 SV=1	P62081	3
178	sp|P28072|PSB6_HUMAN Proteasome subunit beta type-6 OS=Homo sapiens OX=9606 GN=PSMB6 PE=1 SV=4	P28072	3
179	sp|P00747|PLMN_HUMAN Plasminogen OS=Homo sapiens OX=9606 GN=PLG PE=1 SV=2	P00747	3
180	tr|J3KMX3|J3KMX3_HUMAN Alpha-fetoprotein OS=Homo sapiens OX=9606 GN=AFP PE=1 SV=1	J3KMX3 (+1)	3
181	sp|O60701|UGDH_HUMAN UDP-glucose 6-dehydrogenase OS=Homo sapiens OX=9606 GN=UGDH PE=1 SV=1	O60701	3
182	Cluster of tr|E9PC52|E9PC52_HUMAN Histone-binding protein RBBP7 OS=Homo sapiens OX=9606 GN=RBBP7 PE=1 SV=1 (E9PC52)	E9PC52 [4]	3
183	sp|P02649|APOE_HUMAN Apolipoprotein E OS=Homo sapiens OX=9606 GN=APOE PE=1 SV=1	P02649	3
184	Cluster of sp|P15880|RS2_HUMAN 40S ribosomal protein S2 OS=Homo sapiens OX=9606 GN=RPS2 PE=1 SV=2 (P15880)	P15880 [3]	3
185	sp|P49720|PSB3_HUMAN Proteasome subunit beta type-3 OS=Homo sapiens OX=9606 GN=PSMB3 PE=1 SV=2	P49720	3
186	sp|P02765|FETUA_HUMAN Alpha-2-HS-glycoprotein OS=Homo sapiens OX=9606 GN=AHSG PE=1 SV=2	P02765	3
187	sp|P46781|RS9_HUMAN 40S ribosomal protein S9 OS=Homo sapiens OX=9606 GN=RPS9 PE=1 SV=3	P46781	3
188	sp|O00410|IPO5_HUMAN Importin-5 OS=Homo sapiens OX=9606 GN=IPO5 PE=1 SV=4	O00410	3
189	sp|P08670|VIME_HUMAN Vimentin OS=Homo sapiens OX=9606 GN=VIM PE=1 SV=4	P08670	3
190	Cluster of tr|G3XAK1|G3XAK1_HUMAN Hepatocyte growth factor-like protein OS=Homo sapiens OX=9606 GN=MST1 PE=1 SV=1 (G3XAK1)	G3XAK1 [4]	3
191	tr|E9PK25|E9PK25_HUMAN Cofilin-1 OS=Homo sapiens OX=9606 GN=CFL1 PE=1 SV=1	E9PK25 (+3)	3
192	tr|H0Y4R1|H0Y4R1_HUMAN Inosine-5'-monophosphate dehydrogenase 2 (Fragment) OS=Homo sapiens OX=9606 GN=IMPDH2 PE=1 SV=1	H0Y4R1	3
193	sp|Q9NRN5|OLFL3_HUMAN Olfactomedin-like protein 3 OS=Homo sapiens OX=9606 GN=OLFML3 PE=2 SV=1	Q9NRN5	3
194	sp|P01008|ANT3_HUMAN Antithrombin-III OS=Homo sapiens OX=9606 GN=SERPINC1 PE=1 SV=1	P01008	3
195	tr|A0A2R8Y6G6|A0A2R8Y6G6_HUMAN Alpha-enolase OS=Homo sapiens OX=9606 GN=ENO1 PE=1 SV=1	A0A2R8Y6G6 (+1)	3
196	tr|H3BNC9|H3BNC9_HUMAN Uncharacterized protein OS=Homo sapiens OX=9606 PE=3 SV=2	H3BNC9	3
197	tr|A6NMH8|A6NMH8_HUMAN Tetraspanin OS=Homo sapiens OX=9606 GN=CD81 PE=1 SV=1	A6NMH8	3
198	sp|P01266|THYG_HUMAN Thyroglobulin OS=Homo sapiens OX=9606 GN=TG PE=1 SV=5	P01266	3
199	tr|E7EX29|E7EX29_HUMAN 14-3-3 protein zeta/delta (Fragment) OS=Homo sapiens OX=9606 GN=YWHAZ PE=1 SV=1	E7EX29 (+1)	3
200	sp|P62753|RS6_HUMAN 40S ribosomal protein S6 OS=Homo sapiens OX=9606 GN=RPS6 PE=1 SV=1	P62753	3
201	sp|P04004|VTNC_HUMAN Vitronectin OS=Homo sapiens OX=9606 GN=VTN PE=1 SV=1	P04004	3
202	Cluster of sp|P15121|ALDR_HUMAN Aldo-keto reductase family 1 member B1 OS=Homo sapiens OX=9606 GN=AKR1B1 PE=1 SV=3 (P15121)	P15121 [2]	3
203	Cluster of tr|B5MDF5|B5MDF5_HUMAN GTP-binding nuclear protein Ran OS=Homo sapiens OX=9606 GN=RAN PE=1 SV=1 (B5MDF5)	B5MDF5 [2]	3
204	sp|P22234|PUR6_HUMAN Multifunctional protein ADE2 OS=Homo sapiens OX=9606 GN=PAICS PE=1 SV=3	P22234	3
205	sp|P12277|KCRB_HUMAN Creatine kinase B-type OS=Homo sapiens OX=9606 GN=CKB PE=1 SV=1	P12277	3
206	tr|A0A0G2JLD8|A0A0G2JLD8_HUMAN Single-stranded DNA-binding protein, mitochondrial (Fragment) OS=Homo sapiens OX=9606 GN=SSBP1 PE=1 SV=1	A0A0G2JLD8 (+3)	3
207	sp|P31939|PUR9_HUMAN Bifunctional purine biosynthesis protein ATIC OS=Homo sapiens OX=9606 GN=ATIC PE=1 SV=3	P31939	3
208	sp|O75891|AL1L1_HUMAN Cytosolic 10-formyltetrahydrofolate dehydrogenase OS=Homo sapiens OX=9606 GN=ALDH1L1 PE=1 SV=2	O75891	3
209	tr|H0YKD8|H0YKD8_HUMAN 60S ribosomal protein L28 OS=Homo sapiens OX=9606 GN=RPL28 PE=1 SV=1	H0YKD8	3
210	tr|A0A1W2PQ51|A0A1W2PQ51_HUMAN Probable ATP-dependent RNA helicase DDX17 OS=Homo sapiens OX=9606 GN=DDX17 PE=1 SV=1	A0A1W2PQ51 (+1)	3
211	tr|E7EUU4|E7EUU4_HUMAN Eukaryotic translation initiation factor 4 gamma 1 OS=Homo sapiens OX=9606 GN=EIF4G1 PE=1 SV=1	E7EUU4 (+3)	3

212	sp|Q13308|PTK7_HUMAN Inactive tyrosine-protein kinase 7 OS=Homo sapiens OX=9606 GN=PTK7 PE=1 SV=2	Q13308	3
213	sp|Q13332|PTPRS_HUMAN Receptor-type tyrosine-protein phosphatase S OS=Homo sapiens OX=9606 GN=PTPRS PE=1 SV=3	Q13332	3
214	tr|A0A3B3IS80|A0A3B3IS80_HUMAN Fructose-bisphosphate aldolase OS=Homo sapiens OX=9606 GN=ALDOB PE=1 SV=1	A0A3B3IS80 (+1)	3
215	sp|Q9Y4W2|LAS1L_HUMAN Ribosomal biogenesis protein LAS1L OS=Homo sapiens OX=9606 GN=LAS1L PE=1 SV=2	Q9Y4W2	3
216	sp|O60763|USO1_HUMAN General vesicular transport factor p115 OS=Homo sapiens OX=9606 GN=USO1 PE=1 SV=2	O60763	3
217	Cluster of sp|P17858|PFKAL_HUMAN ATP-dependent 6-phosphofructokinase, liver type OS=Homo sapiens OX=9606 GN=PFKL PE=1 SV=6 (P17858)	P17858 [3]	2
218	sp|P04275|VWF_HUMAN von Willebrand factor OS=Homo sapiens OX=9606 GN=VWF PE=1 SV=4	P04275	2
219	sp|P62249|RS16_HUMAN 40S ribosomal protein S16 OS=Homo sapiens OX=9606 GN=RPS16 PE=1 SV=2	P62249	2
220	Cluster of tr|C9JFV4|C9JFV4_HUMAN Proline-, glutamic acid- and leucine-rich protein 1 OS=Homo sapiens OX=9606 GN=PELP1 PE=1 SV=2 (C9JFV4)	C9JFV4 [3]	2
221	Cluster of sp|Q15063|POSTN_HUMAN Periostin OS=Homo sapiens OX=9606 GN=POSTN PE=1 SV=2 (Q15063)	Q15063 [2]	2
222	Cluster of sp|O14744|ANM5_HUMAN Protein arginine N-methyltransferase 5 OS=Homo sapiens OX=9606 GN=PRMT5 PE=1 SV=4 (O14744)	O14744 [2]	2
223	tr|A0A087X054|A0A087X054_HUMAN Hypoxia up-regulated protein 1 OS=Homo sapiens OX=9606 GN=HYOU1 PE=1 SV=1	A0A087X054 (+2)	2
224	Cluster of tr|H9KV75|H9KV75_HUMAN Alpha-actinin-1 OS=Homo sapiens OX=9606 GN=ACTN1 PE=1 SV=1 (H9KV75)	H9KV75 [4]	2
225	Cluster of sp|P23396|RS3_HUMAN 40S ribosomal protein S3 OS=Homo sapiens OX=9606 GN=RPS3 PE=1 SV=2 (P23396)	P23396 [2]	2
226	Cluster of sp|O60506|HNRPQ_HUMAN Heterogeneous nuclear ribonucleoprotein Q OS=Homo sapiens OX=9606 GN=SYNCRIP PE=1 SV=2 (O60506)	O60506 [2]	2
227	sp|P11717|MPRI_HUMAN Cation-independent mannose-6-phosphate receptor OS=Homo sapiens OX=9606 GN=IGF2R PE=1 SV=3	P11717	2
228	sp|Q01518|CAP1_HUMAN Adenylyl cyclase-associated protein 1 OS=Homo sapiens OX=9606 GN=CAP1 PE=1 SV=5	Q01518	2
229	sp|O14980|XPO1_HUMAN Exportin-1 OS=Homo sapiens OX=9606 GN=XPO1 PE=1 SV=1	O14980	2
230	Cluster of sp|P61978|HNRPK_HUMAN Heterogeneous nuclear ribonucleoprotein K OS=Homo sapiens OX=9606 GN=HNRNPK PE=1 SV=1 (P61978)	P61978 [2]	2
231	Cluster of tr|A0A2R8Y7R2|A0A2R8Y7R2_HUMAN Hemoglobin subunit beta OS=Homo sapiens OX=9606 GN=HBB PE=1 SV=1 (A0A2R8Y7R2)	A0A2R8Y7R2 [3]	2
232	Cluster of sp|P62987|RL40_HUMAN Ubiquitin-60S ribosomal protein L40 OS=Homo sapiens OX=9606 GN=UBA52 PE=1 SV=2 (P62987)	P62987 [3]	2
233	sp|P02675|FIBB_HUMAN Fibrinogen beta chain OS=Homo sapiens OX=9606 GN=FGB PE=1 SV=2	P02675	2
234	sp|P62937|PPIA_HUMAN Peptidyl-prolyl cis-trans isomerase A OS=Homo sapiens OX=9606 GN=PPIA PE=1 SV=2	P62937	2
235	Cluster of H7C2U8-DECOY	H7C2U8-DECOY [2]	2
236	sp|P00488|F13A_HUMAN Coagulation factor XIII A chain OS=Homo sapiens OX=9606 GN=F13A1 PE=1 SV=4	P00488	2
237	tr|A0A2R8Y6J3|A0A2R8Y6J3_HUMAN 60S ribosomal protein L5 (Fragment) OS=Homo sapiens OX=9606 GN=RPL5 PE=1 SV=1	A0A2R8Y6J3 (+1)	2
238	sp|O43143|DHX15_HUMAN Pre-mRNA-splicing factor ATP-dependent RNA helicase DHX15 OS=Homo sapiens OX=9606 GN=DHX15 PE=1 SV=2	O43143	2
239	sp|Q86UX7|URP2_HUMAN Fermitin family homolog 3 OS=Homo sapiens OX=9606 GN=FERMT3 PE=1 SV=1	Q86UX7	2
240	sp|O00231|PSD11_HUMAN 26S proteasome non-ATPase regulatory subunit 11 OS=Homo sapiens OX=9606 GN=PSMD11 PE=1 SV=3	O00231	2
241	sp|O15232|MATN3_HUMAN Matrilin-3 OS=Homo sapiens OX=9606 GN=MATN3 PE=1 SV=2	O15232	2
242	Cluster of tr|A0A087WTE4|A0A087WTE4_HUMAN Neural cell adhesion molecule 1 OS=Homo sapiens OX=9606 GN=NCAM1 PE=1 SV=1 (A0A087WTE4)	A0A087WTE4 [5]	2
243	tr|R4GMR5|R4GMR5_HUMAN 26S proteasome non-ATPase regulatory subunit 8 OS=Homo sapiens OX=9606 GN=PSMD8 PE=1 SV=1	R4GMR5	2
244	sp|Q06828|FMOD_HUMAN Fibromodulin OS=Homo sapiens OX=9606 GN=FMOD PE=1 SV=2	Q06828	2
245	sp|P20618|PSB1_HUMAN Proteasome subunit beta type-1 OS=Homo sapiens OX=9606 GN=PSMB1 PE=1 SV=2	P20618	2
246	sp|P43686|PRS6B_HUMAN 26S proteasome regulatory subunit 6B OS=Homo sapiens OX=9606 GN=PSMC4 PE=1 SV=2	P43686	2
247	sp|P05543|THBG_HUMAN Thyroxine-binding globulin OS=Homo sapiens OX=9606 GN=SERPINA7 PE=1 SV=2	P05543	2
248	tr|D6RAR4|D6RAR4_HUMAN Hepatocyte growth factor activator OS=Homo sapiens OX=9606 GN=HGFAC PE=1 SV=1	D6RAR4 (+1)	2
249	sp|Q9NXF1|TEX10_HUMAN Testis-expressed protein 10 OS=Homo sapiens OX=9606 GN=TEX10 PE=1 SV=2	Q9NXF1	2
250	tr|A0A0C4DG17|A0A0C4DG17_HUMAN 40S ribosomal protein SA OS=Homo sapiens OX=9606 GN=RPSA PE=1 SV=1	A0A0C4DG17 (+2)	2
251	sp|P53618|COPB_HUMAN Coatomer subunit beta OS=Homo sapiens OX=9606 GN=COPB1 PE=1 SV=3	P53618	2
252	sp|P54577|SYYC_HUMAN Tyrosine--tRNA ligase, cytoplasmic OS=Homo sapiens OX=9606 GN=YARS1 PE=1 SV=4	P54577	2
253	sp|P32969|RL9_HUMAN 60S ribosomal protein L9 OS=Homo sapiens OX=9606 GN=RPL9 PE=1 SV=1	P32969	2
254	tr|E9PEP6|E9PEP6_HUMAN Neuropilin OS=Homo sapiens OX=9606 GN=NRP1 PE=1 SV=2	E9PEP6 (+1)	2
255	sp|Q8NC51|PAIRB_HUMAN Plasminogen activator inhibitor 1 RNA-binding protein OS=Homo sapiens OX=9606 GN=SERBP1 PE=1 SV=2	Q8NC51	2
256	tr|A0A3B3ITZ9|A0A3B3ITZ9_HUMAN Thyroid hormone receptor-associated protein 3 OS=Homo sapiens OX=9606 GN=THRAP3 PE=1 SV=1	A0A3B3ITZ9 (+1)	2
257	tr|F5GX39|F5GX39_HUMAN Transmembrane emp24 domain-containing protein 2 OS=Homo sapiens OX=9606 GN=TMED2 PE=1 SV=1	F5GX39 (+1)	2
258	tr|C9JC84|C9JC84_HUMAN Fibrinogen gamma chain OS=Homo sapiens OX=9606 GN=FGG PE=1 SV=1	C9JC84 (+1)	2
259	sp|P51665|PSMD7_HUMAN 26S proteasome non-ATPase regulatory subunit 7 OS=Homo sapiens OX=9606 GN=PSMD7 PE=1 SV=2	P51665	2
260	sp|P51570|GALK1_HUMAN Galactokinase OS=Homo sapiens OX=9606 GN=GALK1 PE=1 SV=1	P51570	2
261	sp|P00352|AL1A1_HUMAN Retinal dehydrogenase 1 OS=Homo sapiens OX=9606 GN=ALDH1A1 PE=1 SV=2	P00352	2
262	sp|P37837|TALDO_HUMAN Transaldolase OS=Homo sapiens OX=9606 GN=TALDO1 PE=1 SV=2	P37837	2
263	sp|P63241|IF5A1_HUMAN Eukaryotic translation initiation factor 5A-1 OS=Homo sapiens OX=9606 GN=EIF5A PE=1 SV=2	P63241	2
264	Cluster of tr|C9JIS1|C9JIS1_HUMAN Guanine nucleotide-binding protein G(I)/G(S)/G(T) subunit beta-2 (Fragment) OS=Homo sapiens OX=9606 GN=GNB2 PE=1 SV=1 (C9JIS1)	C9JIS1 [4]	2
265	tr|E9PRY8|E9PRY8_HUMAN Elongation factor 1-delta OS=Homo sapiens OX=9606 GN=EEF1D PE=1 SV=1	E9PRY8	2
266	sp|Q15493|RGN_HUMAN Regucalcin OS=Homo sapiens OX=9606 GN=RGN PE=1 SV=1	Q15493	2
267	sp|O43505|B4GA1_HUMAN Beta-1,4-glucuronyltransferase 1 OS=Homo sapiens OX=9606 GN=B4GAT1 PE=1 SV=1	O43505	2
268	tr|I3L3U9|I3L3U9_HUMAN Ribosomal L1 domain-containing protein 1 (Fragment) OS=Homo sapiens OX=9606 GN=RSL1D1 PE=1 SV=1	I3L3U9 (+1)	2
269	sp|P60174|TPIS_HUMAN Triosephosphate isomerase OS=Homo sapiens OX=9606 GN=TPI1 PE=1 SV=3	P60174	2
270	tr|I3L3H2|I3L3H2_HUMAN Eukaryotic initiation factor 4A-III OS=Homo sapiens OX=9606 GN=EIF4A3 PE=1 SV=2	I3L3H2 (+1)	2
271	sp|P00742|FA10_HUMAN Coagulation factor X OS=Homo sapiens OX=9606 GN=F10 PE=1 SV=2	P00742	2
272	tr|A0A0B4J2B4|A0A0B4J2B4_HUMAN 40S ribosomal protein S15 OS=Homo sapiens OX=9606 GN=RPS15 PE=1 SV=1	A0A0B4J2B4 (+5)	2
273	tr|C9JW96|C9JW96_HUMAN Prohibitin (Fragment) OS=Homo sapiens OX=9606 GN=PHB PE=1 SV=2	C9JW96 (+4)	2
274	sp|O75396|SC22B_HUMAN Vesicle-trafficking protein SEC22b OS=Homo sapiens OX=9606 GN=SEC22B PE=1 SV=4	O75396	2
275	tr|D3YTG3|D3YTG3_HUMAN Target of Nesh-SH3 OS=Homo sapiens OX=9606 GN=ABI3BP PE=1 SV=1	D3YTG3 (+2)	2
276	sp|P04180|LCAT_HUMAN Phosphatidylcholine-sterol acyltransferase OS=Homo sapiens OX=9606 GN=LCAT PE=1 SV=1	P04180	2
277	sp|Q00266|METK1_HUMAN S-adenosylmethionine synthase isoform type-1 OS=Homo sapiens OX=9606 GN=MAT1A PE=1 SV=2	Q00266	2
278	sp|O75131|CPNE3_HUMAN Copine-3 OS=Homo sapiens OX=9606 GN=CPNE3 PE=1 SV=1	O75131	2
279	sp|P62316|SMD2_HUMAN Small nuclear ribonucleoprotein Sm D2 OS=Homo sapiens OX=9606 GN=SNRPD2 PE=1 SV=1	P62316	2
280	sp|P62851|RS25_HUMAN 40S ribosomal protein S25 OS=Homo sapiens OX=9606 GN=RPS25 PE=1 SV=1	P62851	2
281	tr|E9PKZ0|E9PKZ0_HUMAN 60S ribosomal protein L8 (Fragment) OS=Homo sapiens OX=9606 GN=RPL8 PE=1 SV=1	E9PKZ0 (+1)	2
282	Cluster of sp|P05556|ITB1_HUMAN Integrin beta-1 OS=Homo sapiens OX=9606 GN=ITGB1 PE=1 SV=2 (P05556)	P05556 [2]	2

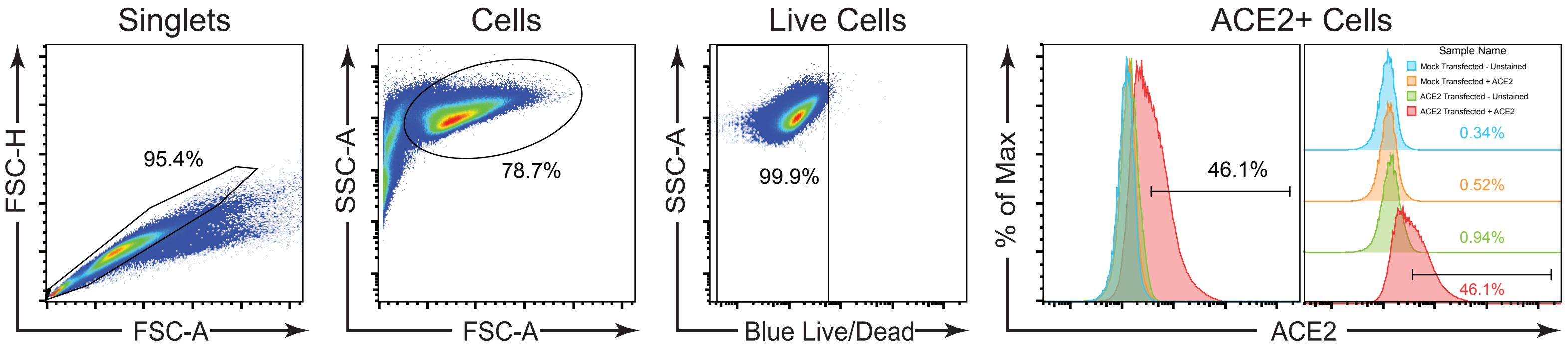

Supplemental Figure 1: Analysis of ACE2 expression in HEK293Ts

Flow cytometry plots showing gating strategy for analysis of ACE2 expression in transiently transfected HEK293Ts. Doublet events were excluded using a FSC-H x FSC-A singlets gate (left plot). Small debris and cell fragments were excluded using a FSC x SSC size gate (middle-left panel) and live cells gated due to their ability to prevent uptake of a LIVE/DEAD marker (middle-right panel). HEK293T cells expressing ACE2 were readily detected by flow cytometry (right panel). ACE2 staining was not detected in mock-transfected HEK293T cells, or when no anti-ACE2 antibody was added.

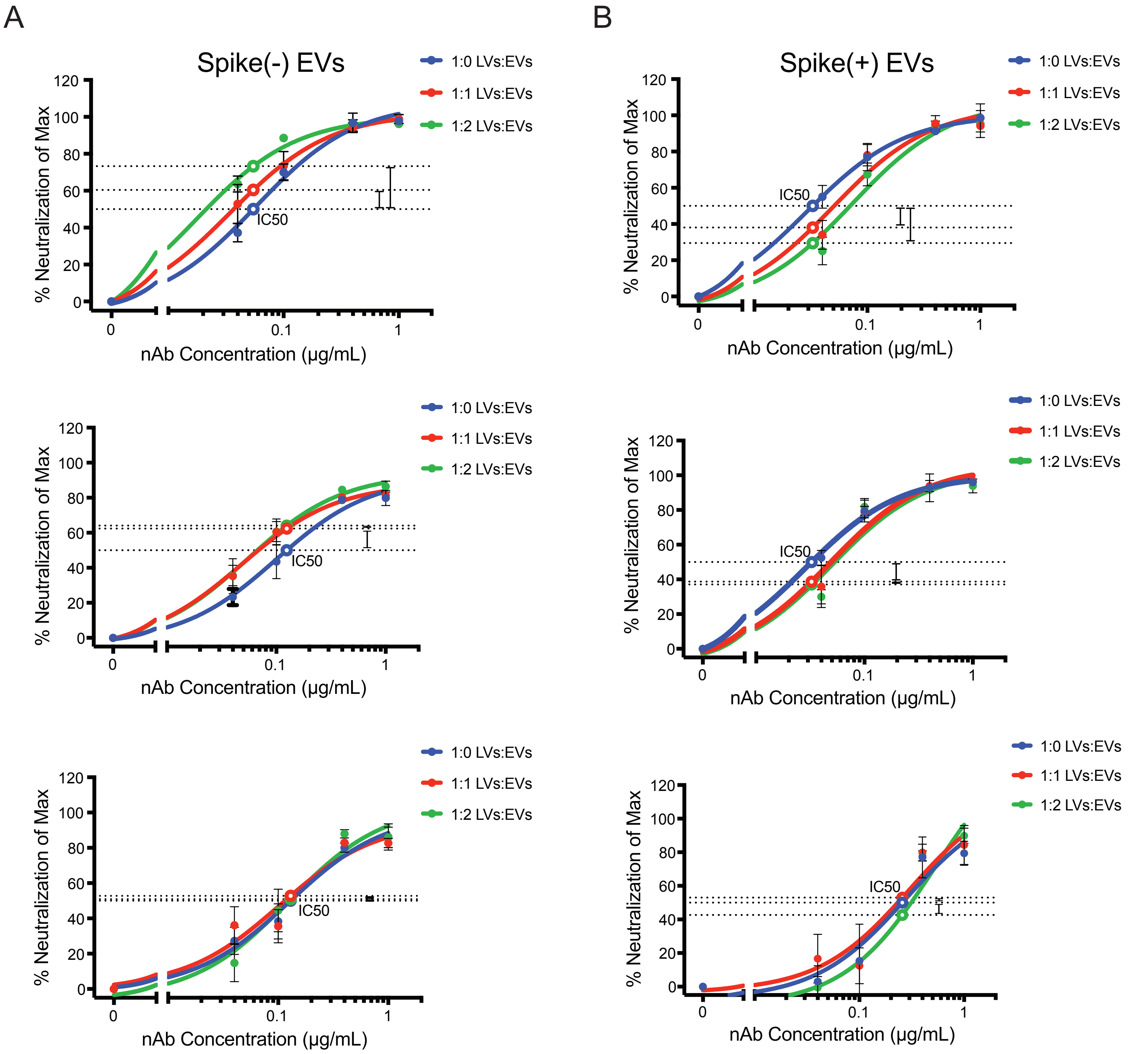

Supplemental Figure 2: Spike(+) EVs reduce the efficiency of commercial neutralizing antibodies at blocking SARS-CoV-2 spike-dependent viral entry – all biological replicates

Dose curves of nAb-mediated inhibition of S-LV entry in the presence of spike(-) EVs (**A**) and spike(+) EVs (**B**) at varying ratios. Each condition was repeated twice to make three independent biological replicates, stacked into columns. Data on each graph are displayed as mean ± SD of technical triplicates. The open point on the blue line (1:0 LVs:EVs) represents the interpolated IC50 value. The open points on the red and green lines represent the interpolated % neutralization value at the IC50 concentration determined from the blue line. Dashed lines are a visual representation of the changes in neutralization efficiency induced by spike(-) or (+) EVs.

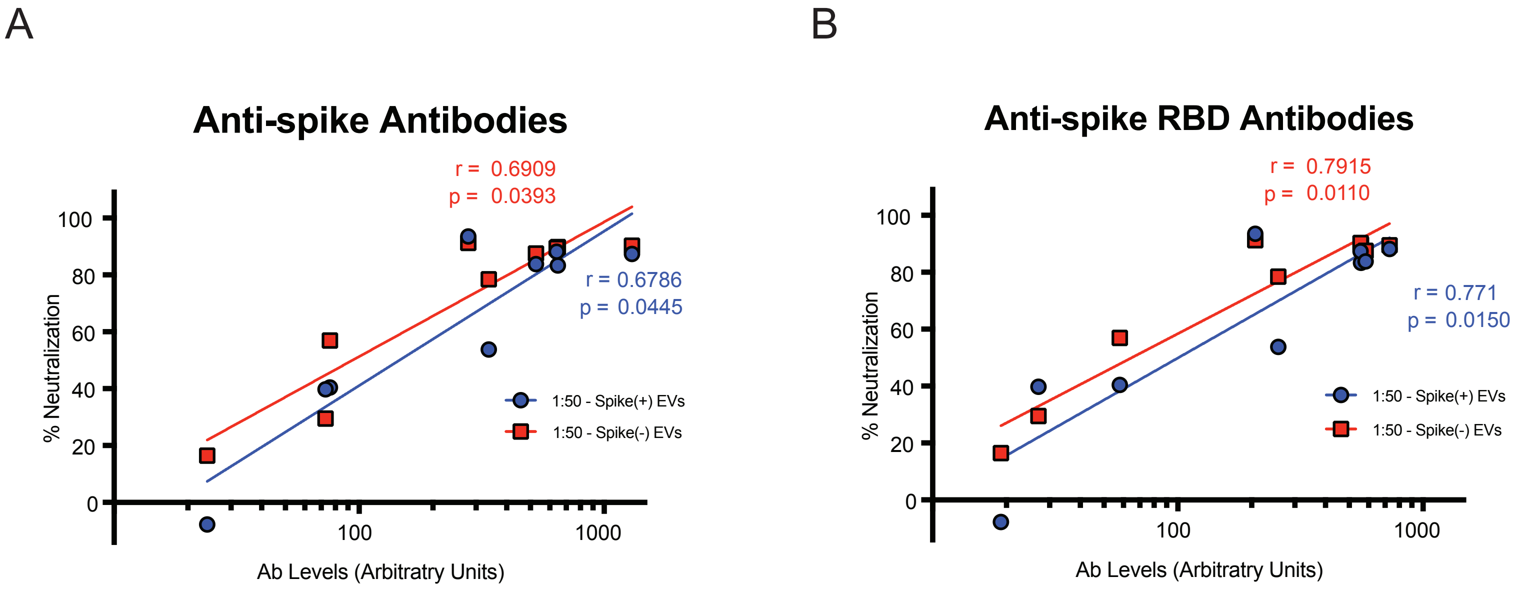

Supplemental Figure 3: Correlations of serum S-LV neutralization values and serum anti-spike antibody levels

Scatter plots representing the relationship between serum neutralization values at a 50-fold dilution and serum anti-spike **(A)** and anti-spike RBD **(B)** antibody titers. The Y-axis represents % neutralization values (100 – normalized % of max infection, as detailed in methods). The X-axis represents anti-spike antibody titers, quantified by arbitrary units. The red symbols represent neutralization in the spike(-) EV condition, while the blue symbols represent the spike(+) EV condition. Pearson correlation was performed in order to determine the relationship between the two variables. A linear regression line was overlaid for each condition in order to represent this correlation. 'r' represents the Pearson correlation coefficient, with a corresponding p-value.

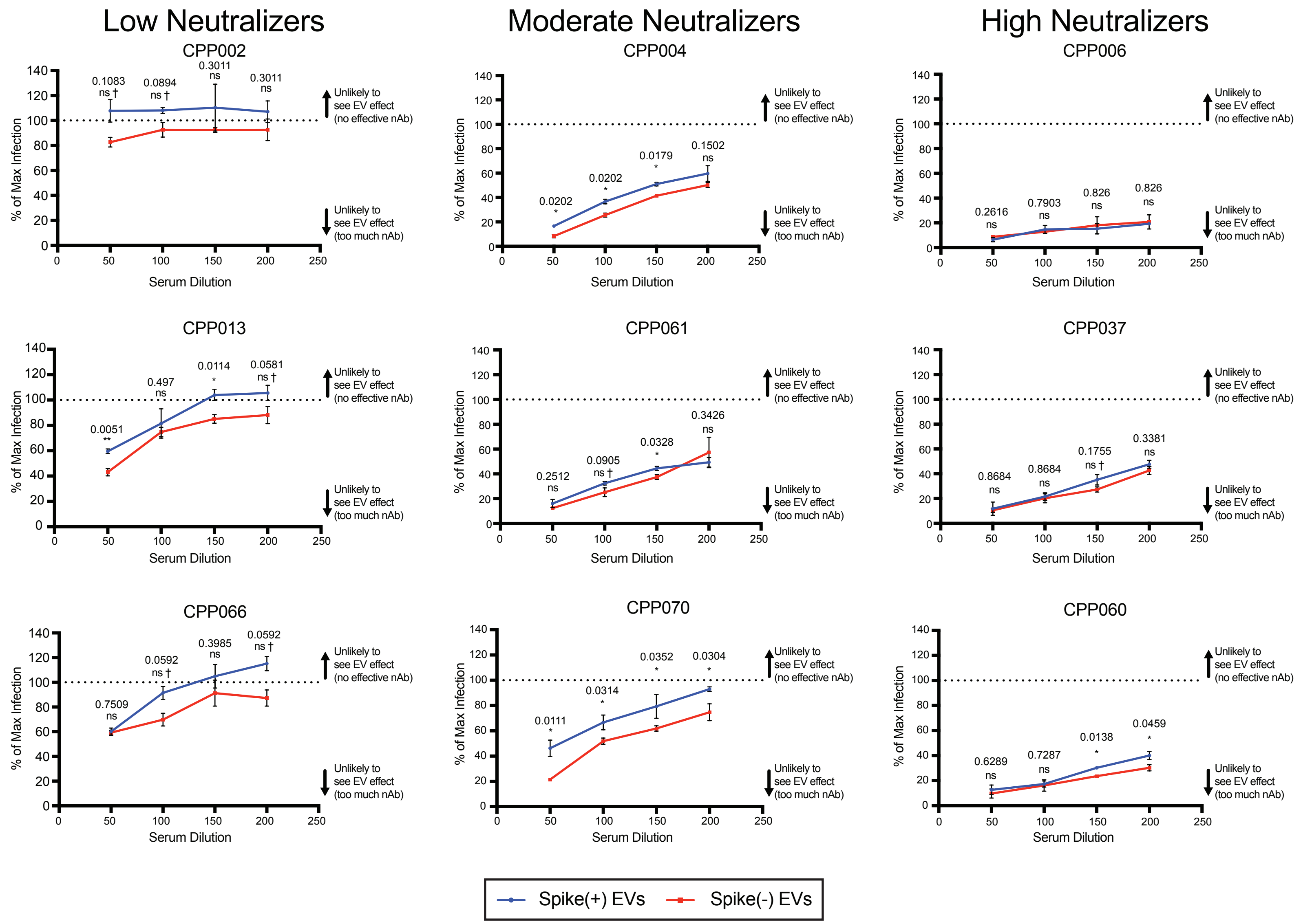

Supplemental Figure 4: Spike(+) EVs reduce the neutralizing ability of convalescent COVID-19 patient sera – all patients

COVID-19 convalescent serum S-LV neutralization curves from 9 different patients representing low, moderate, and high neutralization ability, stacked into corresponding columns. Curves represent serum neutralization in the presence of spike(-) EVs (red) and spike(+) EVs (blue). The Y-axis represents S-LV infection levels normalized to a no serum/no EVs infection control (termed max infection) a calculation described in the methods. The X-axis represents fold-dilution of the patient serum. Data are displayed as mean ± SD of technical triplicates. For patients CPP002, CPP004, and CPP066, the spike(-) EVs data are displayed as mean ± SD of technical duplicates. Significance was measured by multiple T-tests followed by Holm-Sidak correction for multiple comparisons. Asterisks over the columns represent the p-values of the T-tests after correction for multiple comparisons. ns, not significant; ns†, significant prior to correction for multiple comparisons; *, $p \leq 0.05$; **, $p \leq 0.01$.
